# Phenomenology of Subjective Anomalous Experiences in People with Schizophrenia

**DOI:** 10.1007/s11013-026-09989-w

**Published:** 2026-05-23

**Authors:** Orlando Mondragón-Benítez, Lina Díaz-Castro, Fernando Corona-Hernández, Héctor Cabello-Rangel

**Affiliations:** 1 Hospital Psiquiátrico Fray Bernardino Álvarez, Mexico City, Mexico; 2 National Autonomous University of Mexico, Mexico City, Mexico; 3 Instituto Nacional de Psiquiatría, Mexico City, Mexico; 4 National Autonomous University of Mexico, Mexico City, Mexico; 5 Hospital Psiquiátrico Fray Bernardino Álvarez, Mexico City, Mexico

**Keywords:** Psychopathology, Schizophrenia, Subjective phenomena, EAWE interview

## Abstract

Current diagnostic criteria for schizophrenia overlook the patient’s subjective experience, offering a simplified view. To describe the subjective anomalies in the personal experience of the “lived world” in patients diagnosed with schizophrenia via the EAWE interview (Examination of Anomalous World Experience). A qualitative study was conducted between January and May 2024. Semistructured interviews were conducted with patients diagnosed with schizophrenia, using the thematic axes of the EAWE as a framework. Also, the interviews were recorded and transcribed for analysis using ATLAS.ti.v24 software. We interviewed five patients, three men and two women, aged between 18 and 46. The main narrative focused on religious and sexual delusions involving God, the Virgin Mary, or the Holy Spirit, within the context of “Existential Orientation.” Sexual experiences were identified as an emerging category in the interview content. In the “Space and Objects” domain, participants reported hallucinations and blurred vision. The “time and events” domain showed a perception of slowness. The “Other Persons” domain included hypoattunement and paranoia. The “Language” domain revealed disorganized thinking. The “Atmosphere” domain covered déjà vu and emotional emptiness. Conclusions: Individuals may experience their place in the world and their intimate relationships in ways that significantly differ from usual.

**Supplementary Information:**

The online version contains supplementary material available at 10.1007/s11013-026-09989-w.

## Introduction

Schizophrenia is a complex disorder involving genetic, neurobiological, neurochemical, neuropsychological, and environmental factors and is characterized by a set of heterogeneous symptoms (Kahn et al., 2015). It affects approximately 1% of the global population and is one of the leading causes of disability among individuals aged 18 to 44 (Torres Lugo et al., 2022). Diagnosis is grounded in evolving clinical criteria, with the Diagnostic and Statistical Manual of Mental Disorders (DSM) and the International Classification of Diseases (ICD) providing the primary frameworks for its conceptualization (McCutcheon et al., 2020). Symptoms are categorized into three groups, positive, negative, and cognitive, and affect various areas of a patient’s life (Kahn et al., 2015; McCutcheon et al., 2020). Kendler (Kendler, 2016) suggested that this may result in an overemphasis on classification criteria at the expense of a more comprehensive understanding of psychopathology. Phenomenological evaluation proposals focus on the patient’s subjective experience, aiming to understand the prereflective structures that organize their symptoms (Pereira, 2017).

Kendler’s analysis (Kendler, 2016) and the work of Cuesta and Peralta (Cuesta & Peralta, 2016) revealed that there is no linear progression in the evolution of schizophrenia diagnostic criteria but rather a “mosaic of clinical features” with different definitions. Three key findings emerge: (1) modern criteria do not cover all relevant symptoms; (2) they underestimate signs in comparison to symptoms; and (3) they place little emphasis on psychomotor signs. Andreasen (Andreasen, 2006) critiques the DSM for focusing on a “minimal” set of symptoms, which discourages clinicians from delving into the individual’s experience, leading to a dehumanizing approach in psychiatry.

Phenomenology, as a method for analyzing complex realities, is proposed as an alternative. Dörr (2002) argues that phenomenology does not merely describe symptoms but also seeks to understand their essence. This approach has renewed the understanding of schizophrenia, allowing a shift toward a narrative of recovery, where the illness is seen not only as a brain disorder but also in terms of identity and self-meaning (Masedo Gutiérrez, 2021).

Considering mental illness solely as a brain disorder creates a void that gives rise to the assumption that the patient’s experiential alterations are not shaped by the subject’s cultural, religious, or life background. Hence, a contextual approach that reveals how our self-other-world relationships shape our experience is essential (Messas & Fernandez, 2022).

Lived experience as a central element of psychopathological exploration allows clinical decisions to be tailored to the subject’s needs. This gives rise to the importance of understanding the lived world by paying attention to the subject’s environment and context (Pienkos et al., 2023).

The study of the phenomenological psychopathology of the subjective experience of patients with psychosis is essential, yet little research has been done in this area. A systematic review found only 27 studies that evaluated the experience of delusions in the context of psychosis, representing the experience of 373 individuals, all from European or United States populations (Ritunnano et al., 2022).

Given the influence of context on psychopathological experience, documenting patients’ experiences across diverse cultural backgrounds is crucial. Irarrázaval points out that replicating instruments such as EAWE in other clinical samples and cultural settings can provide additional evidence through comparisons across multiple clinical groups and cultures (Irarrázaval, 2020).

In Mexico, there are no studies that address schizophrenia spectrum disorders from a phenomenological perspective using the EAWE instrument; therefore, this study is pioneering in this area. The research question is as follows: What are the subjective anomalies in the personal experience of the “lived world” in patients diagnosed with schizophrenia? The objective of this study was to analyze the phenomenology of subjective experiences in schizophrenia patients via the instrument Examination of Anomalous World Experience (EAWE) (Sass et al., 2017).

## Material and Methods

### Study Design

A qualitative, cross-sectional study with an analytical orientation was conducted between January and May 2024. The study employed a thematically informed approach to explore and understand the subjective experiences of individuals diagnosed with schizophrenia at a specific point in time. Data collection was carried out through semi-structured interviews guided by the conceptual framework of the EAWE.

### Population and Sample

The target population consisted of patients diagnosed with schizophrenia who were receiving treatment at the psychiatric hospital. A theoretical sampling strategy was used in order to ensure diversity in terms of age, sex, and time since diagnosis.

Participants were recruited according to the following inclusion criteria:

- Outpatients receiving treatment in the partial hospitalization program
- Clinical diagnosis of a schizophrenia spectrum disorder
- Age between 18 and 45 years
- Ability to provide informed consent
- No restrictions regarding gender or duration of illness

All participants resided in Mexico City, spoke Spanish as their primary language, and self-identified as mestizo.

### Development of the Interview Guide

A semi-structured interview guide was specifically developed for this study. The guide was constructed using the six domains of the Examination of Anomalous World Experience (EAWE) as predefined thematic axes:

- Space and objects
- Time and events
- Other persons
- Language
- Atmosphere
- Existential orientation

Each domain served as an organizing framework for the formulation of open-ended questions aimed at eliciting detailed descriptions of participants’ lived experiences.

The EAWE was originally developed by Sass et al. (Sass et al., 2017) and published in Psychopathology. For this study, a Spanish-language version adapted to the Mexican context was employed (Hernández, 2020). The interviews were audio-recorded and transcribed verbatim for subsequent analysis.

### Procedure

After preparing the study protocol and obtaining ethical approval, consent was obtained from the head of the External Consultation and Partial Hospitalization Service for recruiting patients.

Eligible patients were invited to participate voluntarily and provided written informed consent prior to the interview.

Interviews were conducted in a private and quiet office within the hospital. When participants showed signs of fatigue or emotional discomfort, the interview was paused and rescheduled. Each interview lasted approximately two hours.

All audio recordings were transcribed verbatim in Spanish and imported into ATLAS.ti version 24.1.0 for systematic qualitative analysis.

### Data Analysis

Data were analyzed using a qualitative content analysis approach with a deductive–inductive orientation, guided by the conceptual structure of the EAWE.

The analysis followed a structured, multi-step process:

Step 1. Definition of Thematic Axes (Deductive Framework)

The six EAWE domains were used as predefined thematic categories. These thematic categories constituted the primary analytical axes for organizing and interpreting the data.

Step 2. Preparation and Organization of Data

Transcripts were reviewed in full and imported into ATLAS.ti (version 24.1.0) for coding and organization. ATLAS.ti is a computer-assisted qualitative data analysis software that facilitates the systematic management of qualitative data, allowing the researcher to assign codes, organize categories, retrieve quotations, and visualize relationships among codes. The software was used exclusively as an analytic support tool; all coding and interpretative decisions were made by the research team.

Step 3. Deductive Coding

In a first cycle of analysis, interview segments were coded deductively according to the thematic categories and their corresponding items and categories. This step ensured that the analysis remained anchored to the phenomenological framework of the instrument.

Step 4. Inductive Coding within Domains

Within each thematic category, an inductive process of open coding was carried out to capture specific experiential nuances expressed by participants. New codes were created whenever relevant aspects of experience were identified that were not fully represented by the predefined thematic categories.

Step 5. Categorization and Organization

Codes were subsequently organized into subcategories and categories within each theme. Patterns, similarities, and variations across participants were identified through constant comparison of coded segments.

Step 6. Integration and Interpretation

Through an iterative analytic process, relationships between codes and categories were examined in order to generate a comprehensive understanding of participants’ subjective experiences. Interpretations were developed linking participants’ narratives to the phenomenological concepts underlying the EAWE.

Step 7. Researcher Triangulation

To enhance the rigor and credibility of the analysis, researcher triangulation was implemented. Two members of the research team (OMB and LDC) independently reviewed and coded the transcripts. Discrepancies in coding or interpretation were discussed in analytic meetings until consensus was reached.

Step 8. Visual Representation of Results

ATLAS.ti was used to generate visual representations of coding patterns (Sankey diagrams). These figures illustrate the distribution of coded quotations within each EAWE domain and serve as qualitative visual support rather than quantitative indicators of symptom severity.

### Ethical Considerations

This study was approved by the Research Committee of the Hospital Psiquiátrico Fray Bernardino Álvarez (registration number: CI-977). Written informed consent was obtained from all participants, who were fully informed about the study’s objectives. The ethical principles established in the Declaration of Helsinki were followed.

## Results

The sample consisted of five participants aged between 25 and 46 years, including three males and two females. Their educational backgrounds range from high school to a master’s degree. The time since symptom onset varies from 7 to 20 years, while the time since receiving a formal diagnosis ranges from 5 to 17 years. All participants were receiving pharmacological treatment, primarily antipsychotic medications such as olanzapine, risperidone, or aripiprazole. See Table 1. The following presents the most relevant findings derived from the semi-structured interviews, organized according to the six domains of the EAWE, used as predefined thematic axes for analysis. Within each thematic axis, categories and subcategories were identified to capture recurring experiential patterns, supported by systematic coding of the data. Categories were considered analytically relevant when they were reported by at least three participants. This criterion allowed the identification of shared patterns while preserving the phenomenological richness of individual accounts, see (Table 2).

**Table 1:** Characteristics of the study participants

Participant identifier	Age	Sex	Education level	Years since symptom onset	Years since diagnosis (and treatment)	Medication	Marital status
BU1	46 years old	Female	High school	20 years	17 years	Olanzapine 15mg/day.	Single
CG2	36 years old	Male	Master’s degree	11 years	8 years	Risperidone 3mg/day.	Single
MA3	31 years old	Male	Incomplete bachelor’s degree	11 years	10 years	Aripiprazole 15mg/day. Escitalopram 10mg/day.	Single
BS4	25 years old	Female	High school	7 years	5 years	Olanzapine 10 mg/day.	Single
RA5	33 years old	Male	Incomplete bachelor’s degree	9 years	7 years	Risperidone 3mg/day. Sertraline 50mg7day.	Single

**Table 2:** Distribution of coded quotations across EAWE domains (used as thematic axes) and emergent categories

	BU1 Gr=206	CG2 Gr=200	MA3 Gr=208	BS4 Gr=193	RA5 Gr=189	Total
1 Space and objects Gr=293; GS=56	58	69	66	59	41	293
2 Time and events Gr=100; GS=32	17	22	19	22	20	100
3 Other persons Gr=239; GS=55	51	50	51	46	41	239
4 Language Gr=137; GS=37	28	25	28	27	29	137
5 Atmosphere Gr=186; GS=60	38	42	41	28	37	186
6 Existential orientation Gr=81; GS=19	18	5	16	19	23	81
Sexual sphere Gr=14; GS=1	6	0	4	0	4	14
Disappearance of people Gr=1; GS=1	0	1	0	0	0	1
Total	216	215	225	201	195	1052

Gr (groundedness) indicates the number of coded quotations associated with each EAWE domain (used as a thematic axis) or emergent category for each participant. GS represents the number of subcategories identified within each domain. The table integrates the coding structure used in the analysis and serves as the basis for the visual representations presented in the figure. Values reflect the distribution of coded experiential data and do not represent quantitative measures of symptom severity.

Domain 1: “Space and Objects” In this domain, all five participants experienced visual illusions and hallucinations: *BU1 1:3 ¶19: “When I was taking a bath, I saw how the foam in the drain took on shapes, figures. They were two angels kissing...”* All the participants also mentioned experiencing visual hallucinations:

MA3 3:15 ¶ 47–48: “... I was watching Eugenio Derbez. Sometimes I talk to the presidents, I mean, I imagine the presidents are greeting me...”

Auditory hallucinations are one of the common items, as all five participants have experienced them. *BU1 1:57 ¶353: “…then I heard two voices very clearly. On one side, it was the Holy Spirit, and on the other, it was Jesus of Nazareth…”*

Another common item within this domain was blurred vision, with four out of the five participants describing this experience: *MA3 3:4 ¶15: “…if I focus my attention on one spot, it feels distorted or blurry, as if it were in motion.”*

Three of the participants experienced tactile disturbances, described as “tingling” or the sensation of “bugs,” resembling formication: *MA3 3:44 ¶153: “…inside the layer of bone, I felt like little ants or spiders, or well, ticklish...”*

Two participants reported other tactile alterations in relation to their delusional experiences. *BU1 1:65 ¶444: “...I clearly felt someone jump on me…,”* which she interpreted as the “Holy Spirit” having sex with her.

**Fig. 1:**
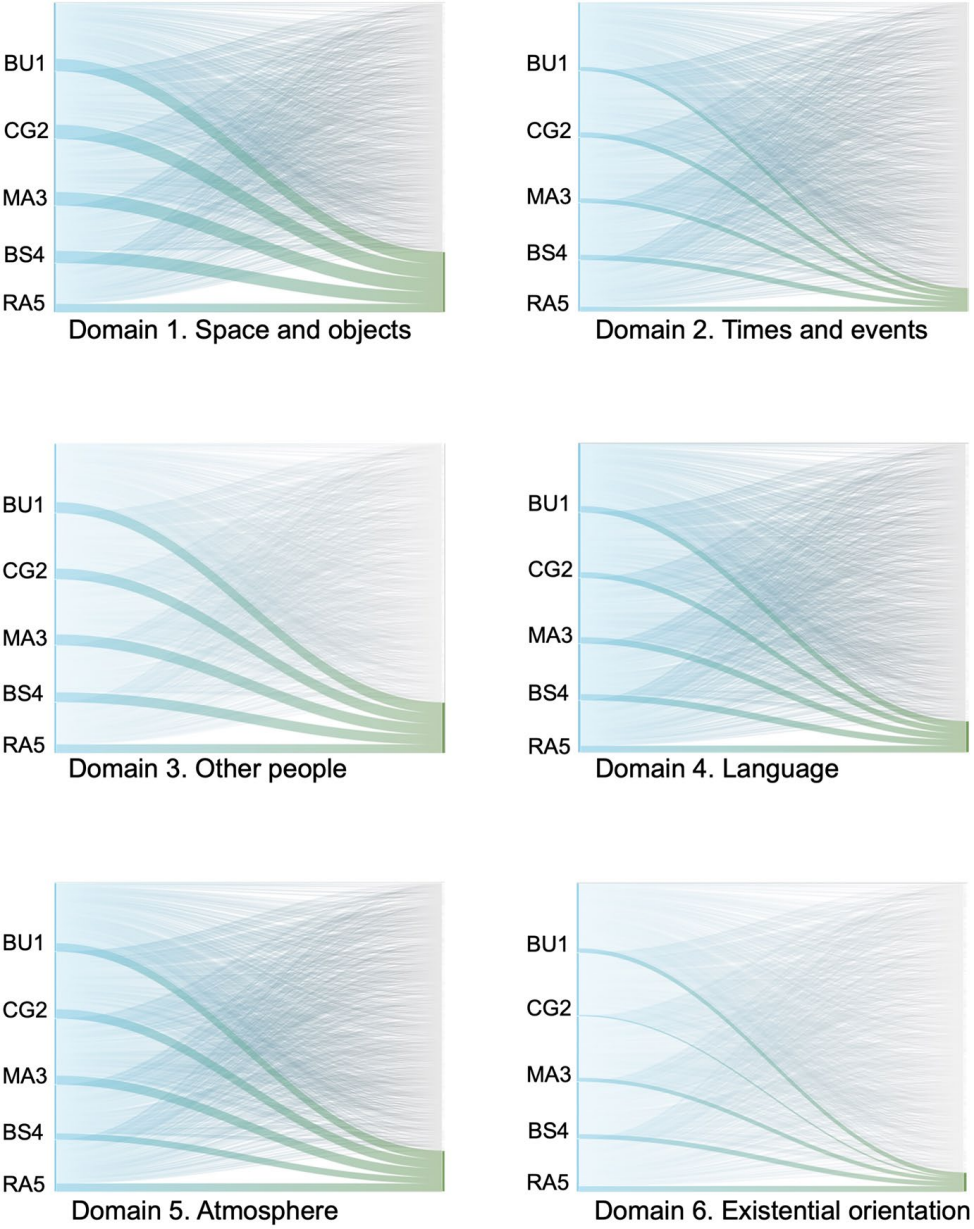
Distribution of coded data across EAWE domains and participants. Sankey diagrams were generated using ATLAS.ti to visualize the distribution of coded excerpts across participants and analytically derived subcategories within each EAWE domain. Each panel corresponds to a distinct domain. Flows represent coded excerpts linking participants (left axis) to subcategories, with widths proportional to their frequency. Colors denote subcategories identified through qualitative content analysis

This domain showed a high density of coded experiential material across participants, as illustrated in Fig. 1 (Domain 1: Space and objects).

Domain 2: “Time and Events” Two of the interviewees experienced various alterations in the perception of “explicit time.” *CG2 2:77 ¶321: “...I felt like I was being tricked about the year, I lost track of the hour, the date, and the month.”*

Within this domain, most patients agreed only on the item “Time or movements seem slowed down,” where three out of five participants experienced alterations in “implicit time,” indicating that the lived time felt slower in relation to the number of activities they engaged in during set periods. For example, BS4 mentioned: *BS4 4:58 ¶272: “It could be that since I’m not doing much or since I’m at home all the time, I don’t work, the week feels very long to me.”*

Compared to other thematic axes, this domain showed a more limited range of experiential variation and a lower density of coded material. These patterns are illustrated in Fig. 1 (Domain 2: Time and events).

Domain 3: “Other Persons” All participants reported alterations in the item “Lack of social understanding or interpersonal attunement (hypoattunement).” They recognized a change in their social common sense, either in the past or currently, they had experienced difficulties in socializing or relating to others. Interviewee CG2 mentioned: *CG2 2:95 ¶375: “As a result of this, I lost confidence. (…) I was terrified of talking to a person. If I talked to you, I would freeze, like when you’re an introvert, but I’ve always been extroverted, so I lost my confidence...”*

These alterations were recognized by the participants as occurring since the onset of their illness or during psychotic crises. During these crises, psychotic symptoms were so distressing that auditory hallucinations conditioned most participants to experience difficulties interacting with others, as well as active social isolation. Regarding this, MA3 commented: *MA3 3:126 ¶425: “I isolated myself a lot in my community because of my hallucinations...”*

Notably, four out of the five participants experienced “Pathological openness.” In the case of MA3, the participant felt exposed, as if their thoughts were revealed without consent: *MA3 3:95 ¶346: “It’s like my imagination was a TV screen where everyone could perceive what I imagined...”*

In this dominion, which was one of the most prominent throughout the interviews, the item of “paranoid significance” emerged, which is related to what is usually referred to as “paranoid delusional ideas.” Within this, several subcategories such as “suspicion of others” and “intrusive of the gazes of others,” were identified. Regarding this, interviewee BU1 mentioned: *BU1 1:118 ¶798: “Yes, I had the feeling that everyone was watching me, like they were trying to destroy me by any means and inform someone else about my movements.”*

As illustrated in Fig. 1 (Domain 3: Other Persons), this category showed a high density of coded material across participants.

Domain 4: “Language” Various alterations in the perception of language were found among the participants, including the use of fillers, word-finding difficulties, difficulty expressing and understanding abstract concepts, and tangential responses. In this last point, it is worth noting that although the participants placed this experience in the past, during their psychotic episodes, frequent derailments and constant tangential responses were observed during the interview. *BS4 4:127 ¶529: “...I try hard to speak, sometimes I can’t find the right words to describe what I want to express.”*

One of the most interesting items was “ Unfocused or Disorganized Thoughts Preclude

Verbal Expression,” where three out of five participants reported realizing this phenomenon. Regarding this, MA3 stated: *MA3 3:151 ¶504: “When doctors interview me, they ask: ‘How did this happen?’ Well, I give a completely different context...”*

As illustrated in Fig. 1 (Domain 4: Language), this domain showed distinct patterns across participants.

Domain 5: “Atmosphere” The participants reported various phenomena related to the atmosphere, including déjà vu experiences, double bookkeeping, self-referentiality, experiences of the end of the world, emotional emptiness, and contradictory and simultaneous mood states. *CG2 2:177 ¶1051: “...Sometimes, suddenly, when I pass by certain houses or streets, I would say to myself, ‘I’ve been here before,’ but no, it’s just a perception...”*

Three out of five participants experienced “Double bookkeeping”: *CG2 2:169 ¶1003: “It was like I was living in two realities...”*

In four interviews, participants had the sense that the world was about to end. Along with this apocalyptic experience, the four interviewees reported feeling a duty or mission to save humanity. *BU1 1:190 ¶1235: “The apocalypse was coming, and I had to sacrifice myself for all of you...”*

Figure 1 (Domain 5: Atmosphere) shows distinct patterns of coded material across participants.

Domain 6: “Existential Orientation” Three of the interviewees experienced a “Feeling of being special or superior,” referring to extraordinary abilities: *BU1 1:202 ¶1343: “I had the gift of speech, command, for example, being perseverant, having willpower, sacrifice, I mean, sacrificing myself for everyone...”*

All five participants referred to having a messianic duty related to the end of the world. RA5, for instance, mentioned receiving a message:

RA5 5:162 ¶696: “...through Jesus, to help saving people because the world was going to end.”

Four out of five participants mentioned having extraordinary intellectual or spiritual abilities. For example, MA3 said: *MA3 3:205 ¶670: “It was like people believed in me, right? They believed in the love that God was sending me because I was special, and they realized that through me, God was very merciful.”*

Three out of five interviewees mentioned new interests, particularly in religion, philosophy, and psychology. Additionally, three participants experienced the sensation of being alienated from or indifferent to what happens on this planet or in reality.

Figure 1 (Domain 6: Existential Orientation) reflects prominent experiential patterns across participants.

Emerging Categories. During the analysis of the semi-structured interviews, two emergent categories were identified: disappearance of people (1 coded quotation) and sexual sphere (14 coded quotations). Given its recurrence and experiential significance across participants, the category related to sexuality was retained for further analysis. See (Table 2).

This category encompassed hallucinations and delusional experiences with sexual content, which are not explicitly captured within the EAWE domains. Its inclusion reflects the presence of clinically and phenomenologically relevant experiences that extend beyond the predefined thematic structure of the instrument.

BU1 1:65 ¶444: “When I was in my room, I mean, the Holy Spirit made me understand that he wanted to have sex with me...”

*MA3 3:86 ¶312: “Well, look, in my hallucinations (...) I thought my community was watching what I was hallucinating. In one of the hallucinations, I had sexual contact with women...”*

## Discussion

This research analyzed the subjective experiences of patients with schizophrenia in various domains of perception, social interaction, language, and existential orientation. A recurring theme among the patients interviewed was religious content in their narratives, God, the Virgin Mary, the Holy Spirit; it is important to note that in Mexico, 80% of the population identifies with a religion, most of them are Catholics or Christians in their many forms (Nacional and de Geografía (INEGI). Panorama de las religiones en México 2020. México: INEGI, c2023.https:, www.inegi.org.mx, app, biblioteca, ficha.htmlupc=^889463910404^. 2020); therefore, the presence of religious content in the interviews constitutes a cultural element worthy of particular attention. Ritunnano found this topic in only three studies out of 27 publications, most of them in European populations. In those publications, the religious theme is the context of integration or support in the community (Ritunnano et al., 2022).

Delusions of religious content are common in patients with schizophrenia. Their presence is related more to deep-rooted cultural factors than to socio-environmental or racial components. A study of the British population found that delusions of religious content were more frequent in people of African and Caribbean origin (Ndetei, 1988).

The theme of religious content varies with the cultural and social environment, and the characteristics of the content may focus on delusions of persecution (by the devil, demonic entities), delusions of grandeur (messianic, special missions), delusions of guilt or devaluation, delusions of passivity or control, and delusions of misidentification. (Sofou et al., 2021) Another study describes the psychopathological processes associated with religious beliefs or delusions, but does not mention sexuality (Drinnan & Lavender, 2006).

We found erotic and sexual elements in the accounts that were not documented in similar studies; the presence of this element may be a consequence of the existential vacuity of the sexual lives of the people studied, and perhaps it is a phenomenon that they wish to preserve in their world, beyond physiological explanations due to the effect of psychotropic drugs on sexual function or difficulties in establishing interpersonal relationships. It can also be assumed to be part of the participants’ existential orientation, in which they experience feelings of superiority, a messianic duty, and extraordinary spiritual abilities, as well as interests in religion, philosophy, and psychology. This may align with what Stanghellini and Ballerini (Stanghellini & Ballerini, 2007) propose as the “axiological dimension” of the disease, where values, principles, and philosophy of life can guide various choices and patterns of behavior, as is the case with most people, with or without a diagnosis of schizophrenia (Pienkos & Sass, 2017a).

Assessing anomalies in the intersubjectivity of schizophrenia is a key element of the EAWE interview, emphasizing the experience of social communication, empathy or lack thereof, interpersonal boundaries of the ego, and the influence or general perception of others (Stanghellini et al., 2017). Anomalies in social understanding were common among the patients interviewed, with all interviewees reporting alterations in the item “Lack of social understanding or interpersonal attunement (hypo-attunement),” which is consistent with the literature. These problems highlight how abnormal experiences can profoundly alter social perception and interaction, affecting how a person relates to the world and others. Alterations in social cognition are present even in the prodromal phase of the disease (Kimoto et al., 2019), significantly affecting interpersonal relationships, employment, school attendance, or independent living (Sergi et al., 2007). However, recent advances in this area remain limited due to ambiguous and inconsistent terminology and differences in measurement approaches (Kimoto et al., 2019).

Analysis of the interviews revealed how temporal and event experiences can vary significantly between individuals, affecting their perception of time, memory, and anticipation of the future. Two of the interviewees experienced various alterations in their perception of “explicit time,” understood as the notion of past, present, and future, where the narrative experience is formed for others and shared with them, allowing us to synchronize our ‘time’ with others (Fuchs & Duppen, 2017). Most agreed with the item “Time or movements seem slowed down”; this experience is related to the lack of activities (recreational and work-related) in patients during certain periods, which could be due to this highly disabling condition, an alteration described by Fuchs and Van Duppen (2017) who; Fuchs & Duppen, cite Eugène Minkowski, who defined the temporal dimension of schizophrenia as a key element in his description of “the loss of vital contact with reality.”

Another element found is pathological openness, an experience similar to “thought transmission,” but differing in that transmission implies agency and the intention to communicate something; agency, understood as the subjective feeling of control or authorship over a particular action or behavior. In the early stages of the disorder, the patient may experience periods of fixation and intention to explore the hallucinations or delusions, which make them more “real” or “vivid,” especially when they or people in their environment pay more attention to them (Jones et al., 2016).

The feeling of greater realism was also present in all patients interviewed when more attention was paid to hallucinations, an experience that has not been widely explored in the literature on schizophrenia. Recent research reports that hallucinations may arise from the confusion of one’s own thoughts with external stimuli due to neurophysiological failures (Whitford et al., 2025). Therefore, these alterations, as part of the patient’s lived world, should be subject to analysis, which may reduce distress and improve quality of life. In our interviews, all participants reported experiencing visual and auditory hallucinations, which is consistent with the findings in the literature, since visual hallucinations are a well-documented phenomenon in patients with schizophrenia, reported in up to 27% of cases, and auditory hallucinations are present in up to 80% of patients with schizophrenia and are a source of significant distress (Font et al., 2003; Silverstein et al., 2017).

Another common symptom is language impairment. In the medical literature on language damage in patients with schizophrenia, there is ongoing debate about whether these disorders represent disturbances in thinking or linguistic ability. Pienkos has described various disturbances in the areas of phonology (e.g., flattened intonation and restricted tone), pragmatics, and lexical access (observed in neologisms and forced speech), although grammar and syntax tend to remain relatively intact (Pienkos & Sass, 2017b). A phenomenological approach to language emphasizes the subjective dimension in these and other linguistic anomalies. In the interviews, various alterations in the participants’ perception of their own language were found, including the use of fillers, lack of words, difficulty expressing and understanding abstract concepts, and tangential responses. It is important to note that, although the interviewees placed this experience in the past during their psychotic episodes, frequent derailments and constant tangential responses were observed during the interview.

One of the most interesting items is “Disorganized thinking that hinders verbal expression.” One might assume that patients with this diagnosis are unaware of their own difficulty in organizing and expressing ideas, but three of the five participants reported being aware of this phenomenon. Detailed clinical descriptions and analyses of the language experience among patients with schizophrenia are rare, so first-person narratives and clinical interviews reveal how people with this disorder encounter and use language (Pienkos & Sass, 2017b).

In our interviews, participants reported various phenomena related to the atmosphere, including déjà vu, double bookkeeping, self-referentiality, experiences of the end of the world, emotional emptiness, and contradictory and simultaneous moods, revealing a wide range of anomalous experiences in the perception of the world and emotions among the interviewees. The items and excerpts from the interviews highlight the subtle but pervasive nature of these changes, which profoundly affect the subjects’ perception of and relationship with their environment. The variety of experiences described underscores the complexity of these anomalies and their impact on people’s daily lives. These anomalies of subjective life have always been central to the phenomenological explanation of mental illness. In fact, Karl Jaspers describes the “delusional mood” as a subtle but generalized disturbance in the general sense of reality and meaning, which often precedes a psychotic episode (Jaspers, 1913; Sass & Ratcliffe, 2017).

On the other hand, the analysis revealed a new emerging category, “sexuality,” a phenomenon that influences patients’ functioning and behavior but has been rarely addressed in similar studies. This approach confirmed the need to incorporate alternative analytical perspectives to find categories or domains other than those already defined, leading to new insights using the EAWE instrument (Irarrázaval, 2020).

Our findings go beyond what has been reported in other studies aimed at describing patients’ sexual activity, (Yang et al., 2023) as they address the sexual content of subjective experience and delirium. Sexuality is a natural component of human behavior and one of the most important elements for quality of life and maintaining satisfying interpersonal relationships (Ma et al., 2018). Furthermore, research indicates that unmet sexual needs in people with schizophrenia can interfere with treatment adherence (Baggaley, 2008). This leads to poor medication adherence and significantly affects their overall quality of life. This issue warrants further investigation due to its critical clinical implications. It should be noted that the influence of daily life and interpersonal functioning on the clinical presentation of schizophrenia underscores the importance of integrated and personalized therapeutic approaches. These interventions, which focus on developing essential skills to improve functional outcomes, may contribute to a better clinical prognosis (Giuliani et al., 2025).

In this study, the experience of psychosis is similar to that reported in studies in high-income countries, except for the recurring narrative of religiosity and the experience of sexuality not reported in previous studies. A systematic review found that the radical reorganization of the lived world dominated by intense emotions, and the search for meaning and coherence are the most significant changes for the patient beyond the mere dysfunction caused by delusions.

Understanding delusional reality allows for greater diagnostic accuracy and therapeutic support for people who, despite treatment, struggle with delusional reality and standard reality, experiences that are not always understood from a medical perspective (Díaz-Castro et al., 2021). This perspective holds that completing a checklist through structured interviews does not imply understanding the patient’s subjective reorganization and perception of reality; these are experiences that persist and worsen during the course of the illness, so that understanding them has implications for psychotherapeutic and pharmacological treatment (Feyaerts et al., 2021; Stephensen et al., 2024).

Phenomenological assessment helps address aspects of the subject’s subjective life, such as the experience of their sexuality, which is sometimes overlooked by clinicians. This is demonstrated by a qualitative phenomenological analysis of sexual needs in which patients expressed that sexual life is essential for building deep relationships and recognized the barriers to exercising it (Yang et al., 2023).

Our study revealed sexuality, which, as mentioned earlier, is rarely addressed from the perspective of the subject’s subjective experience and is a constant concern for patients. This makes it necessary to design meaningful interventions that build confidence and improve skills in intimate relationships (Cloutier et al., 2023).

The study may benefit from including a large and more diverse patient sample, encompassing different stages of schizophrenia and varying severity levels, to explore how anomalous experiences evolve over time. Given the potential for changes in perceptual and cognitive domains over time, longitudinal studies could help track the progression of anomalous experiences and their relationship to clinical outcomes. Anomalous experiences may vary across different cultural and social contexts. Future studies could explore how cultural factors influence the expression and interpretation of these experiences in schizophrenia.

## Conclusions

The study emphasizes the complexity of anomalous subjective experiences in patients with schizophrenia. The findings show that sensory anomalies in “Space and Objects,” such as visual and auditory hallucinations, align with the literature, while the identification of blurry vision and tactile disturbances opens new research directions. Variations in the perception of time and anticipation in the “Time and Events” domain reveal heterogeneous impairments, particularly the sensation of time slowing down.

The “Other Persons” domain highlights social cognition challenges, with hypoattunement, pathological openness, and paranoia affecting interpersonal interactions. Language anomalies, such as disorganized thought and tangential responses, suggest the need for targeted therapeutic strategies. Changes in the “Atmosphere” domain indicate subtle shifts in environmental perception, which may precede psychotic episodes. “Existential orientation” reveals emerging philosophical and religious interests, along with feelings of superiority, suggesting an axiological dimension in schizophrenia. Additionally, the “Sexuality” code emphasizes the need to address sexual disturbances in treatment owing to their impact on quality of life and adherence. Overall, these findings highlight the importance of a phenomenological approach to advance personalized clinical strategies for schizophrenia treatment.

## Supplementary Information

Below is the link to the electronic supplementary material.

Supplementary file1 (PDF 389 KB)

## Data Availability

No datasets were generated or analyzed during the current study.

## Declarations

### Ai Usage Statement

The authors of this document declare that they used AI tools in the adaptation of the bibliographic references to the format established by the journal.
