# Supplementary material for: Phenomenology of Subjective Anomalous Experiences in People with Schizophrenia
Source: Cult Med Psychiatry. 2026 May 23;50(2):33. doi: 10.1007/s11013-026-09989-w (PMC13198455; doi:10.1007/s11013-026-09989-w)
Supplement: Supplementary file 1 — Supplementary file1 (PDF 389 KB) [file 11013_2026_9989_MOESM1_ESM.pdf]

EAWE ítem/subtipo		DESCRIPCIÓN
**1 Espacio y Objetos**		
1.1	*Intensidad y/o persistencia anormal de la percepción visual*	*Colores, luces o imágenes son percibidas con menor o mayor intensidad de la usual, como si no pudieran desvanecerse en el fondo, o que duren más de lo habitual. Esto puede estar asociado con alteración de la sensibilidad a estímulos visuales (aumentada o disminuida).*
1.2	*Visión parcial o total*	*El sujeto experimenta visión borrosa u opaca, o percibe que parte de un objeto presente en su campo de visión falta o no está presente.*
1.3	*Alteraciones en la veracidad de las percepciones visuales*	*El sujeto manifiesta haber percibido erróneamente objetos (haber visto una serpiente en vez de una sombra, por ejemplo), o haber visto cosas que claramente no están ahí. La experiencia puede encontrarse en algún punto intermedio entre ver e imaginar.*
1.4	*Fragmentación visual*	*Un objeto o escena que típicamente se percibe de manera integral y coherente se desintegra o pierde su unidad*
1.5	*Desorganización o perturbación de la estabilidad de los objetos*	*Alteración en la percepción de la integridad u organización de objetos o escenas.*
1.6	*Cambios en la calidad, tamaño o contorno de las percepciones visuales*	*Los objetos se perciben con cambios de forma, color, y tamaño en comparación con cómo los percibe el sujeto normalmente.*
1.7	*Alteraciones de la percepción de la distancia o de la yuxtaposición de los objetos*	*Incluye varias perturbaciones en la estimación de distancias o yuxtaposición de objetos*
1.8	*Distorsión de la experiencia espacial*	*La experiencia de que la estructura general del espacio es inusual o cambió de alguna forma.*
1.9	*Intensidad anormal o persistencia de percepciones auditivas*	*Ruidos y sonidos son escuchados como más o menos intensos de lo usual. Esto puede estar relacionado con mayor o menor sensibilidad a los estímulos auditivos.*
1.10	*Alteraciones en la veracidad o precisión de las percepciones auditivas*	*El sujeto manifiesta haber escuchado mal los sonidos (haber escuchado su nombre en el crujir de una tabla) o escuchar cosas que claramente no están ahí. La experiencia puede ser cuasi-auditiva, en algún punto intermedio entre escuchar y pensar.*
1.11	*Otros cambios en la calidad de las percepciones auditivas*	*El sujeto describe otras transformaciones de diversas cualidades auditivas, por ejemplo, los sonidos pueden percibirse con timbres más agudos o más graves de lo usual (como chillidos o chirridos) o como apagados o distorsionados de alguna otra forma. Esto puede experimentarse con ciertos sonidos o con todos. En caso de que esto sea, principalmente, un tema de entendimiento lingüístico, véase el Dominio 4.*
1.12	*Problemas para localizar sonidos*	*El sujeto tiene dificultad para identificar la fuente de los sonidos, siente confusión de si provienen de la derecha, la izquierda, arriba, abajo, enfrente, detrás, quizá especialmente cuando el origen del sonido no está visible. Nota: no indicarlo aquí si se trata de alucinaciones o pseudoalucinaciones auditivas.*
1.13	*Alteraciones de otros sentidos*	*El sujeto experimenta sensaciones táctiles, gustativas u olfativas como sensaciones diferentes de lo usual.*
1.14	*Sinestesia o percepción concomitante anormal*	*La experiencia de una modalidad sensorial automáticamente causa una respuesta asociada con otra modalidad sensorial. Puede ser una experiencia agradable o disfórica.*
1.15	*Separación o aislamiento de las percepciones sensoriales*	*Los fenómenos sensoriales aparecen como independientes, separados de su origen, como cuando la voz de una persona o el sonido de un animal estuvieran divorciados (separados) de su persona o animal, aunque éste sea visible o se vea emitir el sonido. Esto puede ocurrir con todas las modalidades sensoriales, pero es más frecuente en las percepciones auditivas.*
1.16	*Alteraciones en el reconocimiento o identificación de un objeto percibido*	*El sujeto se siente incapaz (o deficientemente o lentamente capaz) de reconocer o identificar un objeto de una percepción visual o auditiva (que es claramente visible o audible), por ejemplo, de comprender que está viendo una rosa o de reconocer a un conocido, o un sonido como el de un carro frenando o acelerando.*

1.17	*Pérdida de límites o demarcación del mundo físico*	*El sujeto tiene la sensación de ser incapaz de determinar dónde termina su cuerpo y comienza el mundo exterior. Una mezcla física en la que los objetos externos parecen estar dentro o fusionados con el cuerpo del sujeto o viceversa.*
**2 Tiempo y Eventos**		
2.1	*El tiempo o los eventos parecen haber cambiado su velocidad*	*La experiencia instantánea en el tiempo o del movimiento está distorsionada, haciéndola parecer más rápida o más lenta. Generalmente estas alteraciones no están relacionadas con el nivel de actividad de la persona y no deben ocurrir únicamente en los periodos de reposo o actividad*
2.2	*Discrepancia entre el tiempo interno y el externo*	*El sujeto reporta sentir que su reloj interno corre a una velocidad distinta al del resto del mundo. Esto puede experimentarse como un cambio en el tiempo interno, tiempo externo o ambos; lo importante es la discrepancia reportada.*
2.3	*Disrupción de la organización dinámica del tiempo*	*El sujeto siente que el flujo o transcurso normal del tiempo no puede darse por sentado como una medida coherente ni como una medida dinámica de experiencia. Hay una disrupción entre la unidad vivida y viviente o síntesis, donde el presente vivido y experimentado incorpora, en sí mismo, tanto el pasado inmediato (lo que Husserl denomina "retención" o "recuerdo primario") como el futuro anticipado, inmediato (lo que Husserl llama "protensión")*
2.4	*Anticipación alterada*	*La sensación normal de inminencia, de dirección hacia el futuro inmediato anticipado está alterada de alguna manera. El énfasis aquí debe ser en la distorsión de los aspectos de protensión/retención normales de la experiencia temporal.*
2.5	*Perturbación de la conciencia del futuro esperado*	*El futuro imaginado o concebido (en contraposición al futuro inmediato de protensión) se experimenta como irrelevante o inexistente, como altamente amenazador, o como anormalmente conocido o revelado de manera anticipada.*
2.6	*Experiencia alterada de los recuerdos o del pasado*	*El pasado recordado se siente alterado o cambiado de cierta forma, puede ser entrecortado, vago, oscuro, desaparecido, acelerado o demasiado lento, inconexo, o intrusivo*[o]
**3 Otras Personas**		
3.1	*Falta de comprensión social o sintonía interpersonal (hiposintonía)*	*Sensación de distanciamiento o desapego de otros, incluyendo la sensación de que los propios movimientos, gestos o discurso están descoordinados con otras personas, de carecer de sintonía no verbal y en especial de sentido común social (dificultad para entender o adherirse a "las reglas del juego"). Esto puede experimentarse como una pérdida de participación espontánea o resonancia con el mundo social, y/o como autoconsciencia (hiperreflexividad) de la alienación de uno mismo en relación con los otros o con la propia conducta o experiencia. Esto va más allá de la sensación de estar fuera de sincronía con los otros, común en la ansiedad social y la depresión, donde el sentido común social básico se mantiene. No se restringe a personas o situaciones específicas e implica un sentir generalizado de alejamiento (no necesariamente constante) del resto de las personas.*
3.2	*Sensación de lejanía de los demás*	*Sensación de estar separado, alejado, excluido, o profundamente fuera de contacto con otras personas, de no estar involucrado y estar observando a la distancia, como un espectador desapegado, sin sentimientos o conexión emocional espontánea.*
3.3	*Estrategias intelectuales/alienadas para entender a los otros*	*El sujeto se vale de la observación desapegada o racionalizada, y métodos basados en reglas para entender y responder a otros, debido a la sensación de ser incapaz de aprender interacciones sociales comunes.*
3.4	*Sensación de inferioridad, crítica o desconfianza respecto a los demás*	*Dificultad extrema para entablar compenetración/mutualismo o para sentirse seguro con otras personas debido a una sensación de inferioridad o de haber experimentado hostilidad de terceros.*
3.5	*Angustia por inseguridad social generalizada.*	*La simple presencia de otras personas se siente onerosa, altamente estresante, insoportable. Generalmente implica una vulnerabilidad ontológica básica, o inseguridad*

		más basal que la vergüenza, culpa o sensación de inferioridad social como si el sujeto mismo estuviera inestable o vulnerable a ser destruido o aniquilado por los demás. Esto puede conllevar un afecto o emoción angustiante o sensaciones corporales extrañas evocadas por contacto interpersonal.
3.6	*Interferencia por voces*	*El sujeto se siente incapaz de participar normalmente en situaciones sociales debido a que escucha voces (imaginadas o alucinadas) que le distraen o desorganizan.*
3.7	*Alteración de la demarcación Yo - Otros*	*El sujeto siente que su sentido basal de independencia o separación de su ser y otras personas se ha atrofiado o se ha vuelto más fluido de lo normal. Esto puede traer sentimientos inusuales de empatía, apertura, control, fusión o confusión entre el ser propio y los otros, ya sea una experiencia física, psicológica o identitaria.*
3.8	*Dificultades con la mirada*	*Sensaciones de dificultad o incomodidad ante el contacto visual per se. El sujeto nota específicamente el contacto visual o la mirada, se siente intrigado o desconcertado por ello y puede tender a analizarlo.*
3.9	*Despersonalización de los demás*	*Se experimenta a las otras personas como seres mecánicos, ilusorios, irreales o inertes, no como seres humanos vivos. Más de un subtema puede escogerse.*
3.10	*Las personas parecen estar dominadas por una característica única*	*El carácter o la apariencia de otra persona parece estar definida o limitada a una sola característica, como si la persona fuera inseparable de, resumida por completo en, o revelada por la forma de su nariz, o su forma de caminar, o un gesto típico, o un comentario recurrente, por ejemplo.*
3.11	*Aumento en la percepción de la intensidad, vitalidad o realidad de los demás*	*El sujeto percibe a las demás personas como más intensas, vivas, o reales de lo usual, de una manera que parece anormal, extraña o no realmente humana.*
3.12	*Cambios en la cualidad o tonalidad de la apariencia de los demás.*	*La forma como se ven, o la manera en que esa apariencia se experimenta está alterada de una forma extraña.*
3.13	*Pareciera como si las personas estuvieran comunicando algo especial o inusual (más allá de lo obvio)*	*Sensaciones como si otros estuvieran sugiriendo o transmitiendo un mensaje especial, que frecuentemente está dirigido especialmente para el sujeto. Lo que otras personas quieren decir puede estar muy lejos de lo que en realidad dicen. El sujeto puede o no identificar el contenido o significado del mensaje.*
3.14	*Respuestas anómalas, conductuales o actitudes, hacia los demás*	*El sujeto es consciente de actuar, - generalmente se siente obligado a actuar- de maneras inusuales o extraordinarios al relacionarse con otras personas, por ejemplo, retirarse, rebelarse, conformarse, observar, etc., a menudo en asociación con una profunda ansiedad, amenaza interpersonal o perplejidad. Los subtipos pueden tener una cualidad más involuntaria/automática (tal vez catatónica) o más intencional/activa (“antagonómica”; el entrevistador puede encontrar útil investigar sobre esto.*
**4 Lenguaje**		
4.1	*Disrupciones básicas de la comprensión verbal estándar*	*El sujeto experimenta dificultad para entender el discurso hablado o escrito, incluyendo el significado de las palabras, oraciones o secuencias de oraciones. Esto puede ocurrir mientras escucha/lee a otros, pero también al escribir/hablar.*
4.2	*Dificultad para comprender aspectos emocionales o expresivos del habla**	*El sujeto experimenta dificultad para reconocer la entonación emocional en el habla de otros, que puede resultar en una falla en el entendimiento de los matices emocionales en la comunicación.*
4.3	*Cambios específicos en la sensación o el significado de las palabras*	*El sujeto experimenta, y puede usar, palabras en formas anormales, ya sea enfocándose en cualidades físicas de las palabras, asignándole nuevos significados, viendo las palabras como absurdas o arbitrarias o hasta percibiendo que las palabras tienen cierta vida propia.*
4.4	*Uso poco convencional de las palabras, la gramática o el tono o discurso críptico*	*El sujeto usa, se reúsa a dar o ensarta palabras de manera anómala que impiden el entendimiento de otros. Esto a veces puede ser deliberado o cuasi deliberado; el entrevistador debe notar los detalles.*
4.5	*Elocuencia alterada*	*El sujeto está consciente de la dificultad para expresarse debido a una discapacidad o retraso en la fluencia de las palabras, siendo difícil usarlas con precisión o disponibilidad. Algunos otros sienten que su expresión verbal parece inadecuada. El*

		sujeto siente una habilidad disminuida para usar el lenguaje como herramienta para transmitir significados y puede experimentar también estar hiperalerta ante el medio lingüístico.
4.6	*Alteración en la relevancia*	*El sujeto está consciente de los problemas para mantenerse en el curso lineal cuando habla o escribe.*
4.7	*Alteración del compromiso lingüístico o intencionalidad*	*El sujeto está consciente de adoptar o manifestar una manera extraña o estilo de expresión poco común que de alguna manera carece del sentido de compromiso usual emocional o volitivo.*
4.8	*Experiencia anómala de lo abstracto y lo concreto*	*Dificultad para aceptar o entender conceptos generales o abstractos, interrelación anormal entre lo abstracto y lo concreto o enfoque aumentado en lo que es altamente abstracto/general o lo concreto/específico.*
4.9	*Inefabilidad: insuficiencia del lenguaje para describir o expresar (puede incluir la tentación de mantenerse en silencio)*	*El sujeto experimenta el lenguaje como inadecuado o no auténtico de manera profunda, incapaz de describir o expresar lo que realmente importa. Esto puede incluir la inclinación a permanecer en silencio, al menos en ciertos asuntos.*
4.10	*Enajenación de la narrativa personal*	*El sujeto experimenta una profunda sensación de distancia o desconexión cuando se describe a sí mismo o a sus experiencias, como si estuviese hablando de alguien más. Esto no es meramente por el contraste entre el estado emocional presente y las experiencias recordadas, pero parece involucrar una dificultad para identificar a quien se está describiendo, aun cuando se trate de sí mismo.*
**5 Atmósfera**		
5.1	*Desrealización del mundo*	*"Un cambio en la experiencia del entorno: el mundo que rodea al sujeto parece transformado, surreal, extraño, y puede compararse como una película". "Desrealización" es un término ambiguo y amplio; aquí se deberá usar sólo para experiencias que describan un sentido de la inmediatez disminuido, actualidad, utilidad, relevancia, vitalidad, o dinamismo. (Las experiencias de hiperrealismo o de solipsismo se incluyen en temas más adelante). Esto es diferente a una alteración como las alucinaciones o ilusiones: no implica una falla en el reconocimiento la identidad de las personas u objetos, sino la experiencia de un cambio sutil pero penetrante en la sensación, sentido o percepción de la realidad del mundo. El cambio ocurre sin que se pierda la prueba de realidad. Debe anotarse si el cambio ocurre asociado o posterior a un ataque de pánico.*
5.2	*Pérdida de los propósitos (Affordance) de las cosas*	*Los significados prácticos y convencionales de los objetos y eventos desaparecen, reemplazados por una percepción meramente geométrica, visual o estética de las cosas.*
5.3	*Los objetos inanimados parecen vivos o con intencionalidad**	*Objetos o cosas inertes o inanimadas parecen estar vivas, o como si emanaran o estuvieran infundidas con una energía especial que las hace parecer autónomas y vivientes. Puede parecer que los objetos están expresando o comunicando un significado (generalmente al sujeto) de una manera animada o humana.*
5.4	*Incremento en la intensidad o hiperrealismo*	*El sujeto experimenta un aumento generalizado en la intensidad del mundo, no específico a algún modo de percepción sensorial. Los objetos y las cosas solo parecen más llamativos que de costumbre, de alguna forma más intensos y demandantes de la atención del sujeto.*
5.5	*Fenómenos de lo ya visto (Déjà vu)*	*El sujeto menciona experimentar una sensación de familiaridad anormal, tal como que a pesar de que las cosas, situaciones o acontecimientos no han sido vividos o experimentados previamente, éstas parecen familiares. Frecuentemente se sienten más intensas o significativas que la sensación cotidiana de déjà vu.*
5.6	*Fenómenos de lo jamás visto (Jamais vu)*	*El sujeto percibe un objeto, escena, situación o concepto (que sabe que ha encontrado o vivido o experimentado previamente) como si no tuviera experiencia previa de ello, como si lo estuviera viendo, percibiendo o experimentando por primera vez, desconocido y quizá hasta incomprensible.*

5.7	*Perplejidad*	*Sensación profunda y perturbadora de estar perplejo o confundido por el sentido general de la realidad.*
5.8	*Manera anómala de atribuir o percibir significados*	*Hay una cualidad extraña en la manera o el proceso por el cual los objetos en la conciencia experimentan tener un significado o inspiran a que se les asigne significado.*
5.9	*Formas anómalas de significación*	*Las formas anómalas de significado incluyen literalidad, realidad concreta, abstracción, o generalidad inusuales. El significado percibido es anómalo en su forma o estructura, de modo que implica literalidad o realidad concreta, o abstracción o generalidad exageradas.*
5.10	*Concientización intensificada de patrones y tendencias*	*El sujeto es profundamente consciente de patrones y tendencias que por lo general son potencialmente significativos y planeados.*
5.11	*Percepción o interpretación anómala de las relaciones causales*	*Un cambio en el sentido de causa-efecto o de los patrones de eventos, tal que las cosas parecen extrañamente controladas, predeterminadas o planeadas.*
5.12	*"Paranoia" autoconsciente/ontológica generalizada*	*El sujeto tiene una sensación penetrante de estar siendo observado. Esto tiene una cualidad cósmica o particularmente ontológica, como si estuviera siendo observado constantemente por un ser o una consciencia indefinible pero omnipresente, usualmente crítica.*
5.13	*Subjetivismo o independencia ontológica disminuida del mundo experimentado*	*El mundo externo o una parte de él parece carecer de existencia independiente de algún modo, sintiéndose anormalmente dependiente de o restringido a la perspectiva o estado mental del sujeto.*
5.14	*Humor revelatorio o pseudorevelatorio (epifánico)*	*Las cosas parecen tener una calidad indescriptible de distinción, peculiaridad o especialidad; el mundo puede sentirse lleno de significado extraño, de significado misterioso. A pesar de estos sentimientos, el sujeto parece incapaz de comprender o especificar exactamente cuál es el cambio o que podría significar.*
5.15	*Experiencias cuasi místicas*	El sujeto es impactado por un sentido de unidad o por la mera existencia del mundo; esto puede tener una cualidad anímica.
	5.15.1	Unión mística con el mundo
	5.15.2	Experiencia del ser (existencia)
5.16	*Experiencias del fin del mundo*	*La sensación del acontecimiento de la destrucción del mundo o llegando a su fin o su avecinamiento. Puede estar asociado con ciertas percepciones sensoriales, y el sujeto puede llegar a creer en su responsabilidad del evento o que él será el único que lo experimentará.*
5.17	*Anomalías del humor y el afecto.*	*El sujeto experimenta anomalías penetrantes y de tipo anímicas en la sensación afectiva o emocional del mundo, que pueden estar acompañadas de dificultades para percibir, aprehender o expresar emociones normales. Aunque con frecuencia se describen en términos de estados internos, generalmente estos estados de ánimo también implican alteraciones en los sentimientos o en la experiencia del mundo en general.*
**6 Orientación Existencial**		
6.1	*Rechazo a la sociedad o su convencionalismo*	*El sujeto rechaza valores socialmente aceptados o la participación en la sociedad, eligiendo vivir de acuerdo a sus propios valores idiosincráticos o maneras de actuar. Esto suele experimentarse como un gran elemento volitivo; no depende del ánimo alto o bajo o de baja autoestima. Puede estar asociado con sentimientos de inadecuación social o pérdida de la identidad o de la originalidad personal si se identifica muy de cerca con otros o si llega a ser parte de una sociedad.*
6.2	*Indiferencia o aceptación extremas*	*El sujeto muestra una aceptación inusual (respecto a valores o ideas) a una variedad de posibilidades más allá de lo normal, sugiriendo una separación de las preocupaciones normales o del sentido común.*
6.3	*Incredulidad generalizada, escepticismo, o curiosidad acerca de lo obvio o lo dado por sentado*	*Incredulidad persistente o ineludible, dudosa curiosidad acerca de cosas que la mayoría de la gente simplemente da por sentado como una verdad obvia. Esto puede estar ligado*

		a otros patrones de pensamiento o conducta como la necesidad de encontrar la "verdadera" naturaleza del tiempo, vida o del universo.
6.4	*Certeza absoluta*	*El paciente experimenta una sensación de certeza acerca de una interpretación anómala del mundo como si todo fuese tan claro como que 2+2=4, por lo que la evidencia entonces es innecesaria y refutar el concepto es inimaginable. El sujeto expresa una extraordinaria convicción con una incomparable certeza subjetiva, impermeabilidad a otras experiencias o contra argumentos convincentes.*
6.5	*Sensación de ser especial o superior*	*El sujeto experimenta una sensación de ser especial en un sentido extremo, típicamente por la posesión de un conocimiento especial, habilidad, visión o por tener una misión especial o rol que jugar en el mundo o en el universo.*
6.6	*Culpa o responsabilidad imposibles*	*Sentimientos de culpa o de un sobre desarrollado sentido de la responsabilidad por cosas que la persona no tiene la posibilidad de haber adquirido o conseguido, como haber causado una guerra o algún trágico accidente en las noticias o el herir a otros de alguna manera*
6.7	*Sentido de pérdida de la libertad o individualidad*	*El sujeto se experimenta a sí mismo como una especie de autómata que carece de originalidad o libertad a un grado extremo, como si fuese controlado por fuerzas externas.*
6.8	*Adherencia a reglas abstractas, intelectualizadas y/o reglas autónomas*	*El sujeto se siente obligado a seguir de manera estricta una serie de reglas y valores que enfatizan racionalidad, una actitud intelectual, principios abstractos o idealistas, o una necesidad compulsiva de seguir "reglas" o "leyes" idiosincráticas. Esto frecuentemente involucra una ideología intelectual, espiritual, moralista, o utópica, desapegada de la realidad de lo concreto, corpóreo, individual, o contextual de la vida social o práctica.*
6.9	*Cambio existencial o intelectual**	*Interés nuevo o inusual en temas existenciales, filosóficos, metafísicos, religiosos o psicológicos. Los temas más frecuentemente reportados son: fenómenos sobrenaturales, religiosos, experiencias místicas, filosofía, temas trascendentales, meditación, psicología, rituales antiguos, símbolos, reencarnación, la vida después de la muerte, conflicto entre el bien y el mal, comunicación y paz universal, significado de la existencia, el destino de la humanidad, salvación, ciencia no convencional, o temas e ideas relativas a la salud y la nutrición.*
6.10	*El individuo se experimenta a sí mismo como el centro del universo*	*Sentimiento fugaz o persistente de ser el centro del universo, el cual parece estar organizado en torno a, controlado por, o dependiente del sujeto.*
6.11	*El individuo se experimenta a sí mismo como ajeno o fuera del universo*	*El sujeto duda de su propio estado de realidad o del de su mundo inmediato, y cree que, de alguna manera, él existe fuera o separado de otra realidad "más verdadera". El sujeto puede sentir como si él o su entorno son el producto de la imaginación de alguien más, como un personaje de un libro.*

EAWE ítem/subtipo	DESCRIPCIÓN
1 Espacio y Objetos	Los 17 temas del Dominio 1 se refieren a diversas experiencias anómalas del espacio y de los objetos, o de cualidades de percepción sensorial. La mayoría de los temas corresponden a la visión y la experiencia del mundo espacial, pero otras modalidades se incluyen. El enfoque es en aspectos más estables o estáticos del mundo (en oposición a las acciones, fluctuaciones o cambios a lo largo del tiempo)
2 Tiempo y Eventos	Descripción general: los seis temas en el Dominio 2 se refieren a varias maneras en que las acciones, eventos o el paso del tiempo pueden ser experimentados de manera anómala. Aquí el enfoque se centra en la temporalidad y los aspectos dinámicos del mundo, que involucran el movimiento, memoria, anticipación, y cambios graduales. Nótese que dos o más de estos aspectos pueden ocurrir de manera simultánea, y las experiencias de *déjà vu* y *jamais vu* se incluyen en el Dominio 5: atmósfera (entorno).
3 Otras Personas	Descripción General: los 14 temas del Dominio 3 se refieren a experiencias anómalas de otros seres humanos o el mundo interpersonal. El foco aquí son las experiencias de interacción social, empatía o su ausencia, límites del yo interpersonal y en general la perspectiva, influencia o sensación/percepción de otras personas. Las experiencias que se centran en comunicación lingüística (palabras, enunciados, o discurso) se incluyen en el Dominio 4: Lenguaje.
4 Lenguaje	Descripción general: Los 10 temas en el Dominio 4 se refieren a las formas anómalas de experimentar el lenguaje, ya sea el propio o el de otras personas. El enfoque aquí es en la experiencia subjetiva de las palabras y su significado, el flujo o discurso, gramática, conceptos verbales y problemas en la expresión verbal (no en la conducta verbal o estructuras lingüísticas implícitas). Cambios en este dominio pueden ser particularmente difíciles de notar por el propio sujeto en sí mismos. Los entrevistadores pueden llegar a necesitar expresar las preguntas de múltiples formas, fraseando la pregunta inicial en términos generales antes de preguntar por detalles específicos; por ejemplo, en vez de preguntar 4.4. Elección inusual de palabras, gramática, tono, o discurso críptico, uno pudiera preguntar "¿está consciente de querer usar el lenguaje de una manera poco usual?" o "¿le molesta el lenguaje, o se siente inclinado a usarlo de manera diferente que el resto de las personas?".<br><br>Durante la entrevista, se pueden notar ciertas anomalías en el discurso que no están reconocidas o admitidas por el sujeto cuando el tema relevante de la EAWE es explorado. En ese caso, se puede indagar sobre esas anormalidades, pero sólo al final de la entrevista (para no disrumpir el desarrollo y la compenetración con el entrevistado). Si el sujeto sigue negando dichas anomalías, a pesar de haberlas expresado, los entrevistadores deberán anotar la conducta sin otorgarle una calificación en la EAWE (ya que la EAWE evalúa la experiencia subjetiva). Es posible que el entrevistado describa haber tenido experiencias anómalas con el lenguaje, y no responder de manera afirmativa a lo que para la EAWE resulta una pregunta relevante, o no describa la experiencia de manera conceptualmente clara o precisa. En tal supuesto, el entrevistador debe marcar el tema, dado que la descripción del entrevistado sí indica cierto grado de consciencia.
5 Atmósfera	Descripción general; los 17 elementos del Dominio 5 se refieren a las anomalías en las experiencias de las cualidades, sensaciones u organización relativas al mundo exterior. Aquí el hincapié debe recaer en las cualidades sutiles, extrañas o penetrantes que tiene el "horizonte", ambiente, estructura, ánimo, o atmósfera del mundo entero – cómo el sujeto percibe las cosas en general - . Estas características están en todos lados y en ningún lugar; son difíciles de separar o describir por su naturaleza intrínseca. No es necesario que todos los aspectos de la experiencia del sujeto se vean alterados por estos cambios; el énfasis está en lo penetrante o la naturaleza anímica del cambio, que puede fijarse en ciertos objetos o permear a todo el mundo vivido.
6 Orientación Existencial	Los 11 ítems del dominio 6 se refieren a una orientación inusual o una "reorientación fundamental". Las anormalidades en este punto se manifiestan como actitudes, opiniones u orientaciones existenciales. Puede ser útil para obtener mayor información preguntar si el paciente ha mantenido esta orientación existencial con preguntas como ¿"podría decirme hace cuánto o qué tan seguido tiene estos sentimientos de…?" "¿ha tenido estos sentimientos desde que recuerda?, ¿De manera constante o intermitente?", "¿inició esto hasta que tuvo otro tipo de experiencias inusuales que involucren percepciones, pensamientos o sentimientos o tal vez sólo ocurren en presencia de esas otras experiencias, ¿hasta que inició a tomar medicamentos psiquiátricos o algún otro tipo de droga? ¿Hasta que le sucedió alguna experiencia significativa o difícil que cambiara sus circunstancias de vida?"

EAWE ítem/subtipo		DESCRIPCIÓN
**1 Espacio y Objetos**		**Los 17 temas del Dominio 1 se refieren a diversas experiencias anómalas del espacio y de los objetos, o de cualidades de percepción sensorial. La mayoría de los temas corresponden a la visión y la experiencia del mundo espacial, pero otras modalidades se incluyen. El enfoque es en aspectos más estables o estáticos del mundo (en oposición a las acciones, fluctuaciones o cambios a lo largo del tiempo)**
1.1	*Intensidad y/o persistencia anormal de la percepción visual*	*Colores, luces o imágenes son percibidas con menor o mayor intensidad de la usual, como si no pudieran desvanecerse en el fondo, o que duren más de lo habitual. Esto puede estar asociado con alteración de la sensibilidad a estímulos visuales (aumentada o disminuida).*
	1.1.1	Incremento en la intensidad de las percepciones visuales
	1.1.2	Disminución en la intensidad de las percepciones visuales
	1.1.3	Recurrencia o prolongación del estímulo visual
1.2	*Visión parcial o total*	*El sujeto experimenta visión borrosa u opaca, o percibe que parte de un objeto presente en su campo de visión falta o no está presente.*
	1.2.1	Visión borrosa
	1.2.2	Visión parcial
	1.2.3	Visión transitoria
1.3	*Alteraciones en la veracidad de las percepciones visuales*	*El sujeto manifiesta haber percibido erróneamente objetos (haber visto una serpiente en vez de una sombra, por ejemplo), o haber visto cosas que claramente no están ahí. La experiencia puede encontrarse en algún punto intermedio entre ver e imaginar.*
	1.3.1	Ilusiones visuales
	1.3.2	Alucinaciones visuales
	1.3.3	Pseudoalucinaciones visuales
1.4	*Fragmentación visual*	*Un objeto o escena que típicamente se percibe de manera integral y coherente se desintegra o pierde su unidad*
	1.4.1	Fragmentación de los objetos
	1.4.2	Desintegración de una escena
	1.4.3	Fijación de la atención en detalles aislados
1.5	*Desorganización o perturbación de la estabilidad de los objetos*	*Alteración en la percepción de la integridad u organización de objetos o escenas.*
	1.5.1	Desorganización de los contornos de los objetos
	1.5.2	Pérdida de la estabilidad perceptual
1.6	*Cambios en la calidad, tamaño o contorno de las percepciones visuales*	*Los objetos se perciben con cambios de forma, color, y tamaño en comparación con cómo los percibe el sujeto normalmente.*
	1.6.1	Cambios en el color de las percepciones visuales
	1.6.2	Micropsia/Macropsia
	1.6.3	Dismegalopsia
	1.6.4	Metamorfopsia
	1.6.5	Otras distorsiones

1.7	*Alteraciones de la percepción de la distancia o de la yuxtaposición de los objetos*	*Incluye varias perturbaciones en la estimación de distancias o yuxtaposición de objetos*
	1.7.1	Los objetos parecen más cercanos o lejanos (Teleopsia)
	1.7.2	Alteración de la relación espacial (yuxtaposición) de los objetos
	1.7.3	Alteraciones generales en la estimación de la distancia
1.8	*Distorsión de la experiencia espacial*	*La experiencia de que la estructura general del espacio es inusual o cambió de alguna forma.*
	1.8.1	Disminución en la orientación en perspectiva
	1.8.2	Pérdida de la orientación topográfica
	1.8.3	Pérdida de la integridad o estructura espacial
	1.8.4	Pérdida de dimensionalidad
	1.8.5	Experiencia de un espacio infinito
	1.8.6	Inversión de la figura/fondo
	1.8.7	Experiencia afectiva del espacio
1.9	*Intensidad anormal o persistencia de percepciones auditivas*	*Ruidos y sonidos son escuchados como más o menos intensos de lo usual. Esto puede estar relacionado con mayor o menor sensibilidad a los estímulos auditivos.*
	1.9.1	Incremento en la intensidad de las percepciones auditivas
	1.9.2	Disminución de la intensidad de las percepciones auditivas
	1.9.3	Mayor conciencia de las sensaciones auditivas de fondo
	1.9.4	Recurrencia o prolongación de los estímulos auditivos
1.10	*Alteraciones en la veracidad o precisión de las percepciones auditivas*	*El sujeto manifiesta haber escuchado mal los sonidos (haber escuchado su nombre en el crujir de una tabla) o escuchar cosas que claramente no están ahí. La experiencia puede ser cuasi-auditiva, en algún punto intermedio entre escuchar y pensar.*
	1.10.1	Ilusiones auditivas
	1.10.2	Alucinaciones auditivas
	1.10.3	Pseudoalucinaciones auditivas
1.11	*Otros cambios en la calidad de las percepciones auditivas*	*El sujeto describe otras transformaciones de diversas cualidades auditivas, por ejemplo, los sonidos pueden percibirse con timbres más agudos o más graves de lo usual (como chillidos o chirridos) o como apagados o distorsionados de alguna otra forma. Esto puede experimentarse con ciertos sonidos o con todos. En caso de que esto sea, principalmente, un tema de entendimiento lingüístico, véase el Dominio 4.*
1.12	*Problemas para localizar sonidos*	*El sujeto tiene dificultad para identificar la fuente de los sonidos, siente confusión de si provienen de la derecha, la izquierda, arriba, abajo, enfrente, detrás, quizá especialmente cuando el origen del sonido no está visible. Nota: no indicarlo aquí si se trata de alucinaciones o pseudoalucinaciones auditivas.*
1.13	*Alteraciones de otros sentidos*	*El sujeto experimenta sensaciones táctiles, gustativas u olfativas como sensaciones diferentes de lo usual.*
	1.13.1	Alteraciones táctiles
	1.13.2	Alteraciones gustativas
	1.13.3	Alteraciones olfativas

1.14	*Sinestesia o percepción concomitante anormal**	*La experiencia de una modalidad sensorial automáticamente causa una respuesta asociada con otra modalidad sensorial. Puede ser una experiencia agradable o disfórica.*
1.15	*Separación o aislamiento de las percepciones sensoriales*	*Los fenómenos sensoriales aparecen como independientes, separados de su origen, como cuando la voz de una persona o el sonido de un animal estuvieran divorciados (separados) de su persona o animal, aunque éste sea visible o se vea emitir el sonido. Esto puede ocurrir con todas las modalidades sensoriales, pero es más frecuente en las percepciones auditivas.*
1.16	*Alteraciones en el reconocimiento o identificación de un objeto percibido*	*El sujeto se siente incapaz (o deficientemente o lentamente capaz) de reconocer o identificar un objeto de una percepción visual o auditiva (que es claramente visible o audible), por ejemplo, de comprender que está viendo una rosa o de reconocer a un conocido, o un sonido como el de un carro frenando o acelerando.*
1.17	*Pérdida de límites o demarcación del mundo físico*	*El sujeto tiene la sensación de ser incapaz de determinar dónde termina su cuerpo y comienza el mundo exterior. Una mezcla física en la que los objetos externos parecen estar dentro o fusionados con el cuerpo del sujeto o viceversa.*
**2 Tiempo y Eventos**		**Descripción general: los seis temas en el Dominio 2 se refieren a varias maneras en que las acciones, eventos o el paso del tiempo pueden ser experimentados de manera anómala. Aquí el enfoque se centra en la temporalidad y los aspectos dinámicos del mundo, que involucran el movimiento, memoria, anticipación, y cambios graduales. Nótese que dos o más de estos aspectos pueden ocurrir de manera simultánea, y las experiencias de *déjà vu* y *jamais vu* se incluyen en el Dominio 5: atmósfera (entorno).**
2.1	*El tiempo o los eventos parecen haber cambiado su velocidad*	*La experiencia instantánea en el tiempo o del movimiento está distorsionada, haciéndola parecer más rápida o más lenta. Generalmente estas alteraciones no están relacionadas con el nivel de actividad de la persona y no deben ocurrir únicamente en los periodos de reposo o actividad*
	2.1.1	El tiempo o los movimientos parecen acelerados
	2.1.2	El tiempo o los movimientos parecen enlentecidos
	2.1.3	El tiempo y los movimientos parecen (de alguna manera) tanto acelerados como enlentecidos
2.2	*Discrepancia entre el tiempo interno y el externo*	*El sujeto reporta sentir que su reloj interno corre a una velocidad distinta al del resto del mundo. Esto puede experimentarse como un cambio en el tiempo interno, tiempo externo o ambos; lo importante es la discrepancia reportada.*
	2.2.1	El tiempo interno parece más lento que el tiempo común (compartido por todos)
	2.2.2	El tiempo interno parece más rápido que el tiempo común (compartido por todos)
2.3	*Disrupción de la organización dinámica del tiempo*	*El sujeto siente que el flujo o transcurso normal del tiempo no puede darse por sentado como una medida coherente ni como una medida dinámica de experiencia. Hay una disrupción entre la unidad vivida y viviente o síntesis, donde el presente vivido y experimentado incorpora, en sí mismo, tanto el pasado inmediato (lo que Husserl denomina "retención" o "recuerdo primario") como el futuro anticipado, inmediato (lo que Husserl llama "protensión")*
	2.3.1	El tiempo parece haberse detenido por completo, estático, infinito, desaparecido
	2.3.2	El tiempo se percibe como desarticulado o fragmentado
	2.3.3	Desorientación temporal
	2.3.4	Sensación de limitación o aislamiento en el momento presente
	2.3.5	Diversas experiencias extrañas del tiempo
2.4	*Anticipación alterada*	*La sensación normal de inminencia, de dirección hacia el futuro inmediato anticipado está alterada de alguna manera. El énfasis aquí debe ser en la distorsión de los aspectos de protensión/retención normales de la experiencia temporal.*

	2.4.1	Anticipación perpetua	
	2.4.2	Sorpresa constante causada por la incapacidad de anticipar eventos	
	2.4.3	Sensación de que "cualquier cosa puede ocurrir"	
	2.4.4	Colapso de la protensión (Orientación futura)	
2.5	*Perturbación de la conciencia del futuro esperado*		*El futuro imaginado o concebido (en contraposición al futuro inmediato de protensión) se experimenta como irrelevante o inexistente, como altamente amenazador, o como anormalmente conocido o revelado de manera anticipada.*
	2.5.1	El futuro parece inexistente	
	2.5.2	El futuro parece irrelevante o carente de importancia	
	2.5.3	El futuro parece amenazador	
	2.5.4	Premoniciones	
2.6	*Experiencia alterada de los recuerdos o del pasado*		*El pasado recordado se siente alterado o cambiado de cierta forma, puede ser entrecortado, vago, oscuro, desaparecido, acelerado o demasiado lento, inconexo, o intrusivo[o]*
	2.6.1	El Pasado parece desconectado	
	2.6.2	El pasado parece vago u obscuro	
	2.6.3	El pasado desaparece o parece inexistente	
	2.6.4	El pasado parece acelerado	
	2.6.5	El pasado parece enlentecido	
	2.6.6	Intrusividad del pasado	
	2.6.7	Difusión de la distinción entre el pasado y el presente	
	2.6.8	El pasado parece desarticulado	
**3 Otras Personas**		**Descripción General: los 14 temas del Dominio 3 se refieren a experiencias anómalas de otros seres humanos o el mundo interpersonal. El foco aquí son las experiencias de interacción social, empatía o su ausencia, límites del yo interpersonal y en general la perspectiva, influencia o sensación/percepción de otras personas. Las experiencias que se centran en comunicación lingüística (palabras, enunciados, o discurso) se incluyen en el Dominio 4: Lenguaje.**	
3.1	*Falta de comprensión social o sintonía interpersonal (hiposintonía)*		*Sensación de distanciamiento o desapego de otros, incluyendo la sensación de que los propios movimientos, gestos o discurso están descoordinados con otras personas, de carecer de sintonía no verbal y en especial de sentido común social (dificultad para entender o adherirse a "las reglas del juego"). Esto puede experimentarse como una pérdida de participación espontánea o resonancia con el mundo social, y/o como autoconsciencia (hiperreflexividad) de la alienación de uno mismo en relación con los otros o con la propia conducta o experiencia. Esto va más allá de la sensación de estar fuera de sincronía con los otros, común en la ansiedad social y la depresión, donde el sentido común social básico se mantiene. No se restringe a personas o situaciones específicas e implica un sentir generalizado de alejamiento (no necesariamente constante) del resto de las personas.*
	3.1.1	Pérdida del sentido común social	
	3.1.2	Pérdida de sintonía corporal/propiocepción	
	3.1.3	Dificultad específica para entender la comunicación no verbal	

3.2	*Sensación de lejanía de los demás*	*Sensación de estar separado, alejado, excluido, o profundamente fuera de contacto con otras personas, de no estar involucrado y estar observando a la distancia, como un espectador desapegado, sin sentimientos o conexión emocional espontánea.*
3.3	*Estrategias intelectuales/alienadas para entender a los otros*	*El sujeto se vale de la observación desapegada o racionalizada, y métodos basados en reglas para entender y responder a otros, debido a la sensación de ser incapaz de aprender interacciones sociales comunes.*
	3.3.1	Escrutinio alienado de las conductas de los demás
	3.3.2	Aproximación algorítmica para el entendimiento/interacción social
3.4	*Sensación de inferioridad, crítica o desconfianza respecto a los demás*	*Dificultad extrema para entablar compenetración/mutualismo o para sentirse seguro con otras personas debido a una sensación de inferioridad o de haber experimentado hostilidad de terceros.*
	3.4.1	Sentimientos de cohibición y autocrítica incrementada
	3.4.2	Sentimientos de paranoia / ansiedad social
	3.4.3	Desconfianza generalizada de los demás
3.5	*Angustia por inseguridad social generalizada.*	*La simple presencia de otras personas se siente onerosa, altamente estresante, insoportable. Generalmente implica una vulnerabilidad ontológica básica, o inseguridad más basal que la vergüenza, culpa o sensación de inferioridad social como si el sujeto mismo estuviera inestable o vulnerable a ser destruido o aniquilado por los demás. Esto puede conllevar un afecto o emoción angustiante o sensaciones corporales extrañas evocadas por contacto interpersonal.*
3.6	*Interferencia por voces*	*El sujeto se siente incapaz de participar normalmente en situaciones sociales debido a que escucha voces (imaginadas o alucinadas) que le distraen o desorganizan.*
3.7	*Alteración de la demarcación Yo - Otros*	*El sujeto siente que su sentido basal de independencia o separación de su ser y otras personas se ha atrofiado o se ha vuelto más fluido de lo normal. Esto puede traer sentimientos inusuales de empatía, apertura, control, fusión o confusión entre el ser propio y los otros, ya sea una experiencia física, psicológica o identitaria.*
	3.7.1	Hipersintonía
	3.7.2	Influencia inusual sobre los demás
	3.7.3	Apertura patológica
	3.7.4	Experiencias de ser controlado
	3.7.5	Fusión o fluidez de límites psicológicos
	3.7.6	Fusión universal con los demás
	3.7.7	Identidad/actitudes personales inciertas
	3.7.8	Límites físicos inciertos
	3.7.9	Experiencia de ser imitado
3.8	*Dificultades con la mirada*	*Sensaciones de dificultad o incomodidad ante el contacto visual per se. El sujeto nota específicamente el contacto visual o la mirada, se siente intrigado o desconcertado por ello y puede tender a analizarlo.*
	3.8.1	Miradas intrusivas de los demás
	3.8.2	Sentirse expuesto a través de los propios ojos
	3.8.3	La mirada propia es intrusiva

	3.8.4	Deshumanización de los ojos de los demás
	3.8.5	Los ojos como portales cósmicos
	3.8.6	Malestar inespecífico con la mirada
3.9	*Despersonalización de los demás*	*Se experimenta a las otras personas como seres mecánicos, ilusorios, irreales o inertes, no como seres humanos vivos. Más de un subtema puede escogerse.*
	3.9.1	Las personas parecen muertas
	3.9.2	Las personas parecen irreales, falsas o ilusorias
	3.9.3	Las personas parecen mecanizadas
3.10	*Las personas parecen estar dominadas por una característica única*	*El carácter o la apariencia de otra persona parece estar definida o limitada a una sola característica, como si la persona fuera inseparable de, resumida por completo en, o revelada por la forma de su nariz, o su forma de caminar, o un gesto típico, o un comentario recurrente, por ejemplo.*
3.11	*Aumento en la percepción de la intensidad, vitalidad o realidad de los demás*	*El sujeto percibe a las demás personas como más intensas, vivas, o reales de lo usual, de una manera que parece anormal, extraña o no realmente humana.*
3.12	*Cambios en la cualidad o tonalidad de la apariencia de los demás.*	*La forma como se ven, o la manera en que esa apariencia se experimenta está alterada de una forma extraña.*
	3.12.1	Las personas resultan familiares de manera extraña
	3.12.2	Las personas resultan desconocidas de manera extraña
	3.12.3	Las personas parecen disfrazadas
	3.12.4	Las personas parecen extrañamente amenazantes
	3.12.5	Cambios no específicos en la apariencia física de los demás
3.13	*Pareciera como si las personas estuvieran comunicando algo especial o inusual (más allá de lo obvio)*	*Sensaciones como si otros estuvieran sugiriendo o transmitiendo un mensaje especial, que frecuentemente está dirigido especialmente para el sujeto. Lo que otras personas quieren decir puede estar muy lejos de lo que en realidad dicen. El sujeto puede o no identificar el contenido o significado del mensaje.*
	3.13.1	Significación paranoide
	3.13.2	Significación grandiosa
	3.13.3	Significación metafísica
	3.13.4	Significación desconocida
3.14	*Respuestas anómalas, conductuales o actitudes, hacia los demás*	*El sujeto es consciente de actuar, - generalmente se siente obligado a actuar- de maneras inusuales o extraordinarios al relacionarse con otras personas, por ejemplo, retirarse, rebelarse, conformarse, observar, etc., a menudo en asociación con una profunda ansiedad, amenaza interpersonal o perplejidad. Los subtipos pueden tener una cualidad más involuntaria/automática (tal vez catatónica) o más intencional/activa ("antagonómica"; el entrevistador puede encontrar útil investigar sobre esto.*
	3.14.1	Aislamiento social activo*
	3.14.2	Conducta oposicionista y/o negativista*
	3.14.3	Desinhibición social*
	3.14.4	Armonía interpersonal compulsiva*
	3.14.5	Obediencia extrema
	3.14.6	Entretener o divertir a los otros de forma compulsiva*

4 Lenguaje		**Descripción general: Los 10 temas en el Dominio 4 se refieren a las formas anómalas de experimentar el lenguaje, ya sea el propio o el de otras personas. El enfoque aquí es en la experiencia subjetiva de las palabras y su significado, el flujo o discurso, gramática, conceptos verbales y problemas en la expresión verbal (no en la conducta verbal o estructuras lingüísticas implícitas). Cambios en este dominio pueden ser particularmente difíciles de notar por el propio sujeto en sí mismos. Los entrevistadores pueden llegar a necesitar expresar las preguntas de múltiples formas, fraseando la pregunta inicial en términos generales antes de preguntar por detalles específicos; por ejemplo, en vez de preguntar 4.4. Elección inusual de palabras, gramática, tono, o discurso críptico, uno pudiera preguntar "¿está consciente de querer usar el lenguaje de una manera poco usual?" o "¿le molesta el lenguaje, o se siente inclinado a usarlo de manera diferente que el resto de las personas?".**<br><br>**Durante la entrevista, se pueden notar ciertas anomalías en el discurso que no están reconocidas o admitidas por el sujeto cuando el tema relevante de la EAWE es explorado. En ese caso, se puede indagar sobre esas anormalidades, pero sólo al final de la entrevista (para no disrumpir el desarrollo y la compenetración con el entrevistado). Si el sujeto sigue negando dichas anomalías, a pesar de haberlas expresado, los entrevistadores deberán anotar la conducta sin otorgarle una calificación en la EAWE (ya que la EAWE evalúa la experiencia subjetiva). Es posible que el entrevistado describa haber tenido experiencias anómalas con el lenguaje, y no responder de manera afirmativa a lo que para la EAWE resulta una pregunta relevante, o no describa la experiencia de manera conceptualmente clara o precisa. En tal supuesto, el entrevistador debe marcar el tema, dado que la descripción del entrevistado sí indica cierto grado de consciencia.**	
4.1	*Disrupciones básicas de la comprensión verbal estándar*		*El sujeto experimenta dificultad para entender el discurso hablado o escrito, incluyendo el significado de las palabras, oraciones o secuencias de oraciones. Esto puede ocurrir mientras escucha/lee a otros, pero también al escribir/hablar.*
	4.1.1	Disociación entre el significado y el sonido	
	4.1.2	Distracción por posibilidades semánticas	
	4.1.3	Distracción por palabras individuales	
	4.1.4	Dificultades inespecíficas en la comprensión	
4.2	*Dificultad para comprender aspectos emocionales o expresivos del habla**		*El sujeto experimenta dificultad para reconocer la entonación emocional en el habla de otros, que puede resultar en una falla en el entendimiento de los matices emocionales en la comunicación.*
4.3	*Cambios específicos en la sensación o el significado de las palabras*		*El sujeto experimenta, y puede usar, palabras en formas anormales, ya sea enfocándose en cualidades físicas de las palabras, asignándole nuevos significados, viendo las palabras como absurdas o arbitrarias o hasta percibiendo que las palabras tienen cierta vida propia.*
	4.3.1	Centrarse en el sonido o la apariencia de palabras o frases	
	4.3.2	Concepción semántica no convencional, vía significante o fragmentos de palabras	
	4.3.3	Las palabras parecen arbitrarias o absurdas	
	4.3.4	Las palabras o el lenguaje parecen vivos, casi físicos, extrañamente poderosos	
	4.3.5	Referencia lingüística egocéntrica	
4.4	*Uso poco convencional de las palabras, la gramática o el tono o discurso críptico*		*El sujeto usa, se reúsa a dar o ensarta palabras de manera anómala que impiden el entendimiento de otros. Esto a veces puede ser deliberado o cuasi deliberado; el entrevistador debe notar los detalles.*
	4.4.1	Discurso críptico, telegráfico o agramatical	
	4.4.2	Palabras comunes o muletillas	
	4.4.3	Palabras inventadas (neologismos) o uso no convencional de las mismas	
	4.4.4	Manierismos y habla forzada	

4.5	*Elocuencia alterada*	*El sujeto está consciente de la dificultad para expresarse debido a una discapacidad o retraso en la fluencia de las palabras, siendo difícil usarlas con precisión o disponibilidad. Algunos otros sienten que su expresión verbal parece inadecuada. El sujeto siente una habilidad disminuida para usar el lenguaje como herramienta para transmitir significados y puede experimentar también estar hiperalerta ante el medio lingüístico.*
	4.5.1	Falta de palabras / Alogia
	4.5.2	Pensamiento desorganizado que imposibilita la expresión verbal
	4.5.3	Discordancia general entre lo que se pretende expresar y lo expresado
4.6	*Alteración en la relevancia*	*El sujeto está consciente de los problemas para mantenerse en el curso lineal cuando habla o escribe.*
	4.6.1	Descarrilamiento
	4.6.2	Respuestas tangenciales
4.7	*Alteración del compromiso lingüístico o intencionalidad*	*El sujeto está consciente de adoptar o manifestar una manera extraña o estilo de expresión poco común que de alguna manera carece del sentido de compromiso usual emocional o volitivo.*
	4.7.1	Aprosodia (falta de entonación emocional)
	4.7.2	Ecolalia
	4.7.3	El discurso se experimenta como autónomo
4.8	*Experiencia anómala de lo abstracto y lo concreto*	*Dificultad para aceptar o entender conceptos generales o abstractos, interrelación anormal entre lo abstracto y lo concreto o enfoque aumentado en lo que es altamente abstracto/general o lo concreto/específico.*
	4.8.1	Dificultad o desagrado con conceptos abstractos o generales
	4.8.2	Lo abstracto es expresado en términos inusualmente concretos
	4.8.3	Los significados específicos o concretos son expresados en términos inusualmente abstractos o generales
	4.8.4	Discurso vago o muy abstracto
4.9	*Inefabilidad: insuficiencia del lenguaje para describir o expresar (puede incluir la tentación de mantenerse en silencio)*	*El sujeto experimenta el lenguaje como inadecuado o no auténtico de manera profunda, incapaz de describir o expresar lo que realmente importa. Esto puede incluir la inclinación a permanecer en silencio, al menos en ciertos asuntos.*
	4.9.1	El lenguaje resulta insuficiente para expresar experiencias inusuales
	4.9.2	Sensación de que el lenguaje es insuficiente
4.10	*Enajenación de la narrativa personal*	*El sujeto experimenta una profunda sensación de distancia o desconexión cuando se describe a sí mismo o a sus experiencias, como si estuviese hablando de alguien más. Esto no es meramente por el contraste entre el estado emocional presente y las experiencias recordadas, pero parece involucrar una dificultad para identificar a quien se está describiendo, aun cuando se trate de sí mismo.*
**5 Atmósfera**		**Descripción general; los 17 elementos del Dominio 5 se refieren a las anomalías en las experiencias de las cualidades, sensaciones u organización relativas al mundo exterior. Aquí el hincapié debe recaer en las cualidades sutiles, extrañas o penetrantes que tiene el “horizonte”, ambiente, estructura, ánimo, o atmósfera del mundo entero – cómo el sujeto percibe las cosas en general - . Estas características están en todos lados y en ningún lugar; son difíciles de separar o describir por su naturaleza intrínseca. No es necesario que todos los aspectos de la experiencia del sujeto se vean alterados por estos cambios; el énfasis está en lo**

		**penetrante o la naturaleza anímica del cambio, que puede fijarse en ciertos objetos o permear a todo el mundo vivido.**
5.1	*Desrealización del mundo*	*“Un cambio en la experiencia del entorno: el mundo que rodea al sujeto parece transformado, surreal, extraño, y puede compararse como una película”. “Desrealización” es un término ambiguo y amplio; aquí se deberá usar sólo para experiencias que describan un sentido de la inmediatez disminuido, actualidad, utilidad, relevancia, vitalidad, o dinamismo. (Las experiencias de hiperrealismo o de solipsismo se incluyen en temas más adelante). Esto es diferente a una alteración como las alucinaciones o ilusiones: no implica una falla en el reconocimiento la identidad de las personas u objetos, sino la experiencia de un cambio sutil pero penetrante en la sensación, sentido o percepción de la realidad del mundo. El cambio ocurre sin que se pierda la prueba de realidad. Debe anotarse si el cambio ocurre asociado o posterior a un ataque de pánico.*
	5.1.1	Sensación de alejamiento o de que hay una barrera (como si se vieran las cosas a través de un cristal)
	5.1.2	Disminución de la intensidad o sustancialidad
	5.1.3	Desanimación
	5.1.4	Falsedad
	5.1.5	Pérdida de la cualidad atrayente
	5.1.6	Inmovilidad, estaticidad, o intelectualismo mórbido
	5.1.7	Desrealización inespecífica u otras formas de desrealización
5.2	*Pérdida de los propósitos (Affordance) de las cosas*	*Los significados prácticos y convencionales de los objetos y eventos desaparecen, reemplazados por una percepción meramente geométrica, visual o estética de las cosas.*
5.3	*Los objetos inanimados parecen vivos o con intencionalidad**	*Objetos o cosas inertes o inanimadas parecen estar vivas, o como si emanaran o estuvieran infundidas con una energía especial que las hace parecer autónomas y vivientes. Puede parecer que los objetos están expresando o comunicando un significado (generalmente al sujeto) de una manera animada o humana.*
5.4	*Incremento en la intensidad o hiperrealismo*	*El sujeto experimenta un aumento generalizado en la intensidad del mundo, no específico a algún modo de percepción sensorial. Los objetos y las cosas solo parecen más llamativos que de costumbre, de alguna forma más intensos y demandantes de la atención del sujeto.*
5.5	*Fenómenos de lo ya visto (Déjà vu)*	*El sujeto menciona experimentar una sensación de familiaridad anormal, tal como que a pesar de que las cosas, situaciones o acontecimientos no han sido vividos o experimentados previamente, éstas parecen familiares. Frecuentemente se sienten más intensas o significativas que la sensación cotidiana de déjà vu.*
5.6	*Fenómenos de lo jamás visto (Jamais vu)*	*El sujeto percibe un objeto, escena, situación o concepto (que sabe que ha encontrado o vivido o experimentado previamente) como si no tuviera experiencia previa de ello, como si lo estuviera viendo, percibiendo o experimentando por primera vez, desconocido y quizá hasta incomprensible.*
5.7	*Perplejidad*	*Sensación profunda y perturbadora de estar perplejo o confundido por el sentido general de la realidad.*
	5.7.1	Confusión de campos (procesos) mentales
	5.7.2	Interferencia de lo irreal (en los procesos mentales)
	5.7.3	El mundo se experimenta como incoherente o confuso
	5.7.4	Híperconciencia de la dimensión tácita
5.8	*Manera anómala de atribuir o percibir significados*	*Hay una cualidad extraña en la manera o el proceso por el cual los objetos en la conciencia experimentan tener un significado o inspiran a que se les asigne significado.*

	5.8.1	El sujeto impone un significado al objeto	
	5.8.2	El objeto al ser percibido posee un significado inherente (nuevo y especial)	
	5.8.3	Proliferación de significados desde el objeto	
5.9	*Formas anómalas de significación*		*Las formas anómalas de significado incluyen literalidad, realidad concreta, abstracción, o generalidad inusuales. El significado percibido es anómalo en su forma o estructura, de modo que implica literalidad o realidad concreta, o abstracción o generalidad exageradas.*
	5.9.1	Ejemplificación física o literal del significado abstracto	
	5.9.2	Clasificación anómala	
5.10	*Concientización intensificada de patrones y tendencias*		*El sujeto es profundamente consciente de patrones y tendencias que por lo general son potencialmente significativos y planeados.*
5.11	*Percepción o interpretación anómala de las relaciones causales*		*Un cambio en el sentido de causa-efecto o de los patrones de eventos, tal que las cosas parecen extrañamente controladas, predeterminadas o planeadas.*
	5.11.1	Las acciones o eventos parecen estar controladas por una fuerza o voluntad externa	
	5.11.2	Las acciones o eventos parecen estar predeterminadas o planeadas	
5.12	*“Paranoia” autoconsciente/ontológica generalizada*		*El sujeto tiene una sensación penetrante de estar siendo observado. Esto tiene una cualidad cósmica o particularmente ontológica, como si estuviera siendo observado constantemente por un ser o una consciencia indefinible pero omnipresente, usualmente crítica.*
5.13	*Subjetivismo o independencia ontológica disminuida del mundo experimentado*		*El mundo externo o una parte de él parece carecer de existencia independiente de algún modo, sintiéndose anormalmente dependiente de o restringido a la perspectiva o estado mental del sujeto.*
	5.13.1	Subjetivismo/solipsismo	
	5.13.2	Doble registro	
	5.13.3	Influir sobre la realidad física	
	5.13.4	Pseudomovimientos de objetos o personas.	
5.14	*Humor revelatorio o pseudorevelatorio (epifánico)*		*Las cosas parecen tener una calidad indescriptible de distinción, peculiaridad o especialidad; el mundo puede sentirse lleno de significado extraño, de significado misterioso. A pesar de estos sentimientos, el sujeto parece incapaz de comprender o especificar exactamente cuál es el cambio o que podría significar.*
	5.14.1	Particularidad extraña	
	5.14.2	Referencialidad	
		5.14.2a	Significación paranoide*
		5.14.2b	Significación grandiosa
		5.14.2c	Significación metafísica
		5.14.2d	Significado desconocido / inestable
	5.14.3	Extrañeza inexplicable	
5.15	*Experiencias cuasi místicas*		El sujeto es impactado por un sentido de unidad o por la mera existencia del mundo; esto puede tener una cualidad anímica.

	5.15.1	Unión mística con el mundo	
	5.15.2	Experiencia del ser (existencia)	
5.16	*Experiencias del fin del mundo*		*La sensación del acontecimiento de la destrucción del mundo o llegando a su fin o su avecinamiento. Puede estar asociado con ciertas percepciones sensoriales, y el sujeto puede llegar a creer en su responsabilidad del evento o que él será el único que lo experimentará.*
5.17	*Anomalías del humor y el afecto.*		*El sujeto experimenta anomalías penetrantes y de tipo anímicas en la sensación afectiva o emocional del mundo, que pueden estar acompañadas de dificultades para percibir, aprehender o expresar emociones normales. Aunque con frecuencia se describen en términos de estados internos, generalmente estos estados de ánimo también implican alteraciones en los sentimientos o en la experiencia del mundo en general.*
	5.17.1	Vacío, anestesia emocional, indiferencia y falta de respuestas espontáneas hacia el mundo	
	5.17.2	Sensación de bloqueo emocional o afectivo (en el sentido de ser incapaz de expresarlo)	
	5.17.3	Ansiedad generalizada, inespecífica, con miedo a la desaparición	
	5.17.4	Irritación basal, inquietud, enojo (disforia)	
	5.17.5	Euforia desapegada	
	5.17.6	Desmoralización, desesperanza y/o desolación	
	5.17.7	Anormalidades en la constancia del humor	
		5.17.7a	El humor o emoción resulta anormalmente persistente
		5.17.7b	El humor o emoción resulta anormalmente lábil
		5.17.8	Incongruencia afectiva o emocional
		5.17.8a	Afecto o emoción inapropiada para la situación
		5.17.8b	Estados de ánimo o emociones son contradictorios y simultáneos.
**6 Orientación Existencial**		**Los 11 ítems del dominio 6 se refieren a una orientación inusual o una "reorientación fundamental". Las anormalidades en este punto se manifiestan como actitudes, opiniones u orientaciones existenciales. Puede ser útil para obtener mayor información preguntar si el paciente ha mantenido esta orientación existencial con preguntas como ¿"podría decirme hace cuánto o qué tan seguido tiene estos sentimientos de…?" "¿ha tenido estos sentimientos desde que recuerda?, ¿De manera constante o intermitente?", "¿inició esto hasta que tuvo otro tipo de experiencias inusuales que involucren percepciones, pensamientos o sentimientos o tal vez sólo ocurren en presencia de esas otras experiencias, ¿hasta que inició a tomar medicamentos psiquiátricos o algún otro tipo de droga? ¿Hasta que le sucedió alguna experiencia significativa o difícil que cambiara sus circunstancias de vida?"**	
6.1	*Rechazo a la sociedad o su convencionalismo*		*El sujeto rechaza valores socialmente aceptados o la participación en la sociedad, eligiendo vivir de acuerdo a sus propios valores idiosincráticos o maneras de actuar. Esto suele experimentarse como un gran elemento volitivo; no depende del ánimo alto o bajo o de baja autoestima. Puede estar asociado con sentimientos de inadecuación social o pérdida de la identidad o de la originalidad personal si se identifica muy de cerca con otros o si llega a ser parte de una sociedad.*
	6.1.1	Aversión por la sociedad	
	6.1.2	Antagonomía	
	6.1.3	Idionomía	

6.2	*Indiferencia o aceptación extremas*	*El sujeto muestra una aceptación inusual (respecto a valores o ideas) a una variedad de posibilidades más allá de lo normal, sugiriendo una separación de las preocupaciones normales o del sentido común.*
	6.2.1	Actitud indiferente, desconsiderada o despreocupada
	6.2.2	Libertad conceptual o "todo se vale"
6.3	*Incredulidad generalizada, escepticismo, o curiosidad acerca de lo obvio o lo dado por sentado*	*Incredulidad persistente o ineludible, dudosa curiosidad acerca de cosas que la mayoría de la gente simplemente da por sentado como una verdad obvia. Esto puede estar ligado a otros patrones de pensamiento o conducta como la necesidad de encontrar la "verdadera" naturaleza del tiempo, vida o del universo.*
6.4	*Certeza absoluta*	*El paciente experimenta una sensación de certeza acerca de una interpretación anómala del mundo como si todo fuese tan claro como que 2+2=4, por lo que la evidencia entonces es innecesaria y refutar el concepto es inimaginable. El sujeto expresa una extraordinaria convicción con una incomparable certeza subjetiva, impermeabilidad a otras experiencias o contra argumentos convincentes.*
6.5	*Sensación de ser especial o superior*	*El sujeto experimenta una sensación de ser especial en un sentido extremo, típicamente por la posesión de un conocimiento especial, habilidad, visión o por tener una misión especial o rol que jugar en el mundo o en el universo.*
	6.5.1	Sentimiento de una visión extraordinaria (en la dimensión escondida de la realidad de la mente, con un sentido especial intelectual o poderes creativos)
	6.5.2	Deber mesiánico
	6.5.3	Grandiosidad intelectual o espiritual
6.6	*Culpa o responsabilidad imposibles*	*Sentimientos de culpa o de un sobre desarrollado sentido de la responsabilidad por cosas que la persona no tiene la posibilidad de haber adquirido o conseguido, como haber causado una guerra o algún trágico accidente en las noticias o el herir a otros de alguna manera*
6.7	*Sentido de pérdida de la libertad o individualidad*	*El sujeto se experimenta a sí mismo como una especie de autómata que carece de originalidad o libertad a un grado extremo, como si fuese controlado por fuerzas externas.*
6.8	*Adherencia a reglas abstractas, intelectualizadas y/o reglas autónomas*	*El sujeto se siente obligado a seguir de manera estricta una serie de reglas y valores que enfatizan racionalidad, una actitud intelectual, principios abstractos o idealistas, o una necesidad compulsiva de seguir "reglas" o "leyes" idiosincráticas. Esto frecuentemente involucra una ideología intelectual, espiritual, moralista, o utópica, desapegada de la realidad de lo concreto, corpóreo, individual, o contextual de la vida social o práctica.*
6.9	*Cambio existencial o intelectual**	*Interés nuevo o inusual en temas existenciales, filosóficos, metafísicos, religiosos o psicológicos. Los temas más frecuentemente reportados son: fenómenos sobrenaturales, religiosos, experiencias místicas, filosofía, temas trascendentales, meditación, psicología, rituales antiguos, símbolos, reencarnación, la vida después de la muerte, conflicto entre el bien y el mal, comunicación y paz universal, significado de la existencia, el destino de la humanidad, salvación, ciencia no convencional, o temas e ideas relativas a la salud y la nutrición.*
6.10	*El individuo se experimenta a sí mismo como el centro del universo*	*Sentimiento fugaz o persistente de ser el centro del universo, el cual parece estar organizado en torno a, controlado por, o dependiente del sujeto.*

6.11	*El individuo se experimenta a sí mismo como ajeno o fuera del universo*	*El sujeto duda de su propio estado de realidad o del de su mundo inmediato, y cree que, de alguna manera, él existe fuera o separado de otra realidad "más verdadera". El sujeto puede sentir como si él o su entorno son el producto de la imaginación de alguien más, como un personaje de un libro.*
